# The Discovery of Endo-Fucanases in the GH141 Family: A Novel Functional Activity Within the Family

**DOI:** 10.3390/ijms27010443

**Published:** 2025-12-31

**Authors:** Nikita Konstantinovich Rubtsov, Artem Sergeevich Silchenko, Marina Petrovna Isaeva, Roman Alekseevich Shkrabov, Anastasiya Olegovna Zueva, Mikhail Igorevich Kusaykin, Svetlana Pavlovna Ermakova

**Affiliations:** 1 Laboratory of Enzyme Chemistry, G.B. Elyakov Pacific Institute of Bioorganic Chemistry, Far-Eastern Branch of the Russian Academy of Sciences, 159, Prospect 100-Let Vladivostoku, 690022 Vladivostok, Russia; rubtsov.nk@yandex.ru (N.K.R.); shkrabov.ra@outlook.com (R.A.S.); zstasya95@gmail.com (A.O.Z.); mik@piboc.dvo.ru (M.I.K.); svetlana_ermakova@hotmail.com (S.P.E.); 2 Laboratory of Marine Biochemistry, G.B. Elyakov Pacific Institute of Bioorganic Chemistry, Far-Eastern Branch of the Russian Academy of Sciences, 159, Prospect 100-Let Vladivostoku, 690022 Vladivostok, Russia; issaeva@gmail.com

**Keywords:** the GH141 family, fucose-containing sulfated polysaccharides, fucoidans, endo-fucanase, fucoidan-degrading gene cluster, fucoidan degradation, transglycosylation

## Abstract

Brown algae produce structurally complex sulfated fucose-containing polysaccharides known as fucoidans. These compounds are slowly degraded by marine microorganisms, leading to their accumulation in marine sediments and contributing to long-term carbon sequestration. The enzymatic mechanisms underlying fucoidan degradation remain poorly understood. GH141 family enzymes are widely distributed among fucoidan-degrading bacteria, but their function remains hypothetical. It is assumed that during fucoidans degradation, they may act as α-L-fucosidases. We performed a biochemical and bioinformatic analysis of four recombinant enzymes, Wf141_1, Wf141_2, Wf141_3, and Wf141_4, of the GH141 family from the fucoidan-degrading cluster of the marine bacterium *Wenyingzhuangia fucanilytica* CZ1127^T^. Sequence similarity network (SSN) and Conserved Unique Peptide Pattern (CUPP) analysis of the GH141 members revealed that the Wf141s enzymes are distant from previously characterized GH141 members and belong to separate SSN clusters and CUPP branches. All four enzymes exhibited endo-fucanase activity against (1→3;1→4)-α-L-fucoidans. Wf141_1 and Wf141_2 were characterized as sulfated (1→3;1→4)-α-L-fucan endo-1→4-α-L-fucanases (EC 3.2.1.212) with distinct substrate preferences: Wf141_1 preferred [→3-α-L-Fucp2S-1→4-α-L-Fucp2S-1→]_n_ fragments, whereas Wf141_2 favored [→3-α-L-Fucp2S-1→4-α-L-Fucp2,3S-1→]_n_ regions. Their specificity depends on structural differences in sugar-binding subsites that recognize sulfation patterns. These enzymes were classified as endo-1→4-α-L-fucanases (EC 3.2.1.212). These findings establish a previously uncharacterized fucoidan-degrading enzymatic function within the GH141 family.

## 1. Introduction

Fucoidans are a class of fucose-rich sulfated polysaccharides produced by brown algae. Brown algal species produce structurally heterogeneous polysaccharides that differ markedly in several key aspects: the types and proportions of monosaccharide units (including sulfated fucans, galactofucan variants, and fucogalactan structures), the arrangement of glycosidic bonds (both α- and β-configurations) linking individual residues, and the nature of chemical modifications, particularly sulfate groups, acetyl groups, and branched structures [1,2,3]. Beyond brown algae, polysaccharides with similar fucose-based backbones occur in marine fauna and microorganisms. Notable examples include the protective gel layer surrounding sea urchin gametes, which contains sulfated fucose polymers [4,5], the integument of sea cucumbers, which exhibits both fucose sulfate and fucosylated chondroitin sulfate components [6,7], and specific diatom species [8,9].

Recently, fucoidans and other fucoidan-related polysaccharides have been shown to have an extremely low turnover rate, which promotes carbon dioxide (CO_2_) sequestration in deeper waters and sediments [8,9,10]. The polymer chains of fucoidans, decorated with various carbohydrate (branching) and non-carbohydrate (sulfation and acetylation) substituents, make these polysaccharides difficult targets for degradation by marine microorganisms. Only highly specialized marine bacteria are able to effectively degrade these complex polysaccharides [10]. Brown algae convert gigatons of carbon dioxide annually into carbohydrates such as fucoidans. Therefore, these unique polysaccharides and fucoidan-degrading marine microorganisms play an important role in the global CO_2_ balance [11].

The known-to-date, fucoidan-degrading marine bacteria produce a vast number of hydrolytic enzymes, represented mainly by glycoside hydrolases of the families GH29, GH95, GH107, GH141, and GH168, as well as sulfatases of the families S1_15, S1_16, S1_17, S1_22, and S1_25 [10,12,13,14,15,16]. The enzymes of these families are often organized into clusters or polysaccharide utilization loci (PUL) and, in many cases, are up-regulated in the presence of fucoidans [10,12,16,17]. Notably, many genes found in fucoidan-degrading PUL of marine bacteria have not been functionally characterized and classified into specific GH [10].

Despite recent advances in genomic and metagenomic analysis, the degradation of fucoidans by microbial enzymes remains poorly understood. It is assumed the backbone of fucoidans (homopolysaccharides) is depolymerized into short oligosaccharides by endo-acting fucanases specific for α-1→3- (EC 3.2.1.211) or α-1→4-glycosidic linkages (3.2.1.212) between sulfated L-fucose residues. To date, endo-fucanase activity has been found among members of the GH107, GH168, GH174, and GH187 families [18] [http://www.cazy.org/ (accessed on 1 November 2025)]. Some members of these families are known to exhibit strict substrate specificity regarding the glycosidic linkage type of the polysaccharide backbone [17,19]. For instance, the GH107 endo-fucanase P5AFcnA from *Psychromonas* sp. exclusively cleaves α-1→3-glycosidic bonds within fucoidans and/or fucooligosaccharides composed of repeating α-1,3-linked sulfated α-L-fucose residues, showing no activity against backbones with alternating α-1→3- and α-1→4-linkages [20]. In addition to their specificity for glycosidic linkage types, these enzymes also discriminate between distinct sulfation patterns in fucoidans. For example, certain members of the GH107 family specifically hydrolyze α-1→4-glycosidic bonds between 2-sulfated L-fucose residues, while others recognize more complex motifs consisting of alternating 2-sulfated and 2,3-di- or 2,4-di-sulfated L-fucose residues [13,21]. This specificity enables the generation of a broader structural spectrum of oligosaccharides as reaction products.

The resulting oligosaccharides, upon entering the bacterial periplasm, are subjected to further degradation into monosaccharides and sulfate, with the participation of exo-acting α-L-fucosidases (EC 3.2.1.51, EC 3.2.1.111, EC 3.2.1.63, and EC 3.2.1.127) and sulfatases of the S1 family. α-L-Fucosidase activity has now been found among the GH29, GH95, GH139, GH141, and GH151 families [http://www.cazy.org/ (accessed on 1 November 2025)]. α-L-Fucosidases can be involved in the debranching of fucoidans, which could potentially lead to more efficient depolymerization of the fucoidan backbone with endo-fucanases. Recent studies have confirmed that fucoidan-active α-L-fucosidases of the GH29 and GH95 families, together with the exo-sulfatases of the S1_17 and S1_25 families, are involved in the step-wise degradation of sulfated fucooligosaccharides and are able to release the L-fucose residues from fucoidans [14]. It is assumed that enzymes of the GH141 family, similar to the GH29 and GH95, may act as exo-acting fucosidases during fucoidan degradation [8,10,12,22]. This assumption is based on the observation of α-L-fucosidase activity in the GH141 member BT1002, which acts on pectic polysaccharides—substrates that are structurally distinct from fucoidans [23]. Consequently, the specific function of GH141 family enzymes in the context of fucoidan degradation remains unconfirmed and requires further investigation.

Members of the GH141 family have been found in most of the identified fucoidan-degrading PULs of known marine bacteria [8,10,12,22,24]. Therefore, members of this family constitute a core pathway dedicated to fucoidan degradation. However, the role of GH141 family enzymes in the degradation of fucoidans remains unknown.

According to the CAZY database (December 2025), the GH141 family includes more than one thousand amino acid sequences. But, only two members of this family have been characterized: endo-1→4-β-D-xylanase Xyn141E, isolated from the bacterium *Acetivibrio thermocellus* ATCC 27405 (basionym: *Clostridium thermocellum*), and α-L-fucosidase BT1002, isolated from the human gut bacterium *Bacteroides thetaiotaomicron* VPI-5482. Xylanase Xyn141E was shown to be an endo-type enzyme that cleaves β-1,4-glycosidic linkages within an arabinoxylan backbone [25]. An unusual α-L-fucosidase BT1002 was identified during a study of the enzyme complex of the bacterium *B. thetaiotaomicron* VPI-5482 that degrades the pectic polysaccharides [23]. This enzyme was found to release L-fucose residues substituted at O-3 with the 2-*O*-Me-α-D-xylose residue from rhamnogalacturonan-II. All these data indicate that the GH141 family is multifunctional and its members can be specific for different types of glycans. Obviously, the mode of action of the GH141 enzymes on glycan molecules can also be different, being either exo- or endo-. Therefore, the functional role of the GH141 family members can be different depending on the glycan type and needs to be further investigated.

The present work describes the bioinformatic and biochemical analysis of four enzymes, Wf141s Wf141_1, Wf141_2, Wf141_3, and Wf141_4 (further short notation is Wf141s), of the GH141 family from the fucoidan-degrading cluster of the marine bacterium *Wenyingzhuangia fucanilytica* CZ1127^T^. Conserved Unique Peptide Pattern (CUPP) and sequence similarity network (SSN) analysis of their amino acid sequences, as well as previously characterized xylanase Xyn141E and α-L-fucosidase BT1002, showed that they constitute separate branches and clusters of the GH141 family. Wf141_1, Wf141_2, and Wf141_4 belong to the GH141*6 CUPP subfamily, while Wf141_3 contains the GH141:4.1 domain and possibly belongs to the GH141*2 CUPP branch. It was found that all of Wf141s lack α-L-fucosidase activity, but possess endo-fucanase activity, cleaving fucoidan molecules into short sulfated fucooligosaccharides. The specificity of two endo-fucanases, Wf141_1 and Wf141_2, was investigated in detail using fucoidans and fucooligosaccharides of different structures in tandem with nuclear magnetic resonance (NMR) spectroscopy. Both enzymes were shown to cleave α-1→4-glycosidic linkages between sulfated L-fucose residues (EC 3.2.1.212), but to recognize different sulfation patterns in fucoidans. Homologues of Wf141_1 and Wf141_2 were mainly found in marine bacteria of the genera *Formosa*, *Algoriphagus*, and *Mariniflexile*.

The data obtained provide the first evidence for a novel functional activity of the GH141 family and its role in fucoidan degradation. These data will allow us to reconsider the mechanisms of fucoidan degradation by marine bacteria.

## 2. Results and Discussion

**Figure 1:**
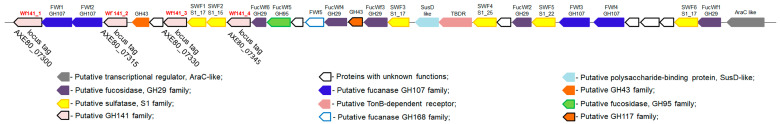
The fucoidan-degrading cluster of the genome of *W. fucanilytica* CZ1127^T^. The enzymes of the GH141 family are indicated in red text.

The genome of the fucoidan-degrading marine bacterium *W. fucanilytica* CZ1127^T^ was previously sequenced (GenBank: GCA_001697185.1). A gene cluster involved in fucoidan degradation was previously identified [13,15]. Several enzymes from this cluster were characterized in detail [13,14,15,16,21,26]. The cluster includes, among others, four enzymes that belong to the GH141 family. Members of this family are widely distributed in some fucoidan-degrading bacteria [10]; however, the role of the GH141 family enzymes in fucoidan degradation are still remains unclear. The presence of these enzymes in the fucoidan-degrading locus may indicate their direct involvement in fucoidan degradation. In the present work, these enzymes, designated as Wf141_1 (GenBank: ANW96096.1), Wf141_2 (GenBank: ANW96099.1), Wf141_3 (GenBank: WP_068825880.1), and Wf141_4 (GenBank: ANW97461.1), were used to study their functional activity (Figure 1).

### 2.1. Amino Acid Sequence Analysis of Wf141s

**Figure 2:**
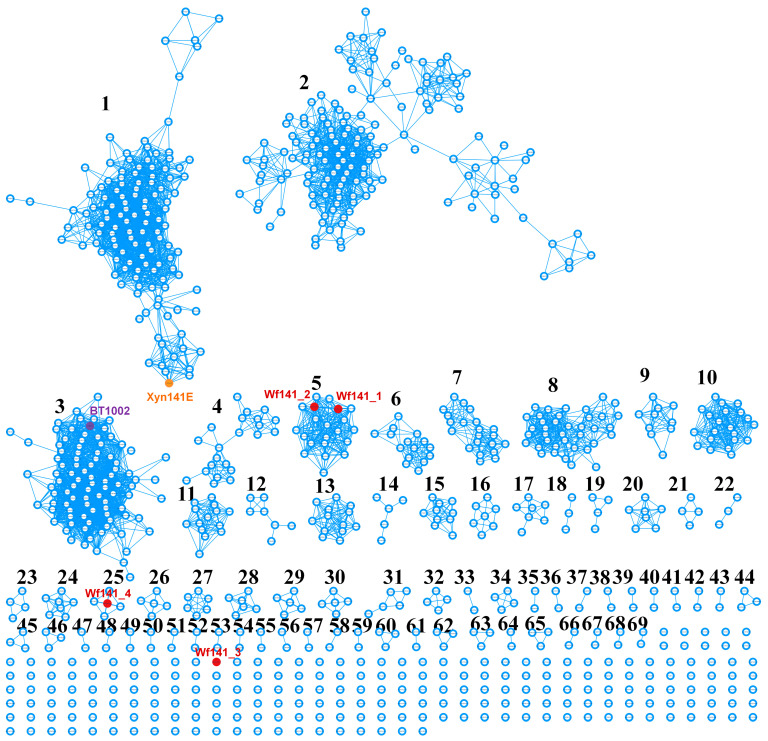
The distribution of the Wf141s and functionally characterized members of the GH141 family among generated SSN clusters. Wf141_1 (cluster 5), Wf141_2 (cluster 5), Wf141_3 (singleton), and Wf141_4 (cluster 25) are colored in red, while α-L-fucosidase BT1002 (cluster 3) and endo-1→4-β-D-xylanase Xyn141E (cluster 1) are colored in purple and orange.

Sequence similarity networks (SSNs) are a widely used approach for elucidating relationships among protein sequences and have proven effective in organizing proteins into functionally related families [27,28,29]. In order to elucidate the structure–function relationships between the enzymes Wf141s of the marine bacterium *W. fucanilytica* and other GH141 family members deposited in the CAZY database (July 2024), we employed the SSN clustering of their sequences. The obtained SSN consisted of the 69 clusters and 216 singletons (Figure 2). The enzymes Wf141s and the previously characterized α-L-fucosidase BT1002 and the endo-1→4-β-D-xylanase Xyl141E are distributed in different clusters, reflecting their different functional activities. The sequences of Wf141_1 and Wf141_2 are grouped into one cluster (cluster 5), while Wf141_4 (cluster 25) and Wf141_3 (singleton) are grouped into two different clusters. This observation may indicate a potential difference in their specificities and/or specific activities. Clusters 5 and 25 can be associated with the fucoidan degradation, but further functional verification is needed to confirm this statement.

**Figure 3:**
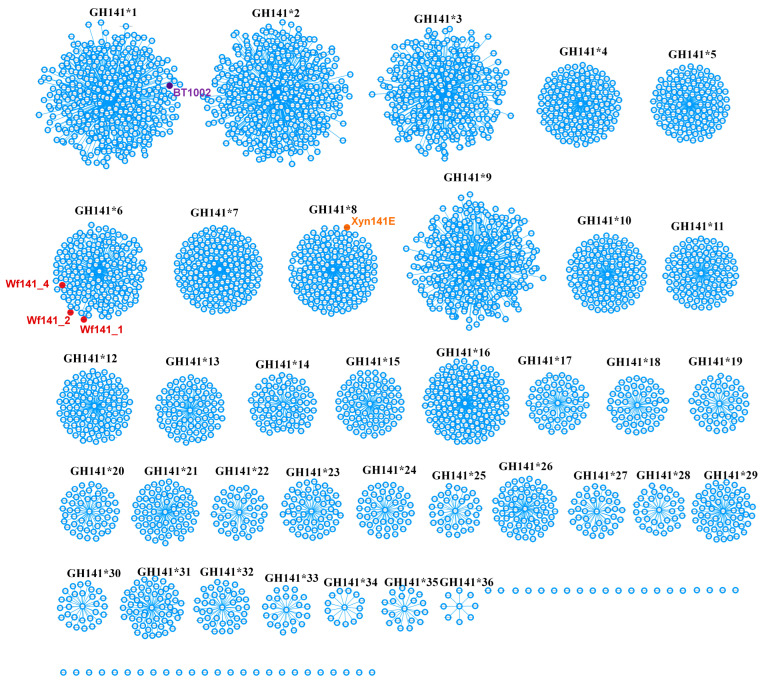
The distribution of Wf141_1, Wf141_2, Wf141_4, and functionally characterized members of the GH141 family among branches predicted by CUPP in the GH141 family. Wf141_1, Wf141_2, and Wf141_4 (branch GH141*6) are colored in red while α-L-fucosidase BT1002 (branch GH141*1) and endo-1→4-β-D-xylanase Xyn141E (branch GH141*8) are colored in purple and orange, respectively.

Among recent innovations in CAZyme identification, the Conserved Unique Peptide Pattern (CUPP) methodology demonstrates enhanced detection capability relative to sequence alignment-based strategies, including Hidden Markov Model (HMM) profiling, InterProScan analysis, and dbCAN searches, which rely on comprehensive protein sequence comparisons [30]. According to the CUPP database, the GH141 family is currently (June 2024) divided into 36 branches. CUPP analysis shows that the Wf141_1, Wf141_2, and Wf141_4 belong to the GH141*6 branch of the GH141 family (Figure 3). Analysis of the distribution of representatives of the GH141*6 branch in the NCBI and CUPP databases revealed their presence in the genomes of marine bacteria, Formosa algae, *Formosa haliotis*, *Lentimonas* sp. CC4, *Lentimonas* sp. CC10 and *Flavobacterium algicola*, known for their fucoidan-degrading ability, as well as *Mariniflexile* sp. AS56, and *Algoriphagus* sp. KMM 8435 (Figure S1) [10,31,32,33]. Therefore, this branch of the GH141 family can be associated with the degradation of fucoidans.

**Figure 4:**
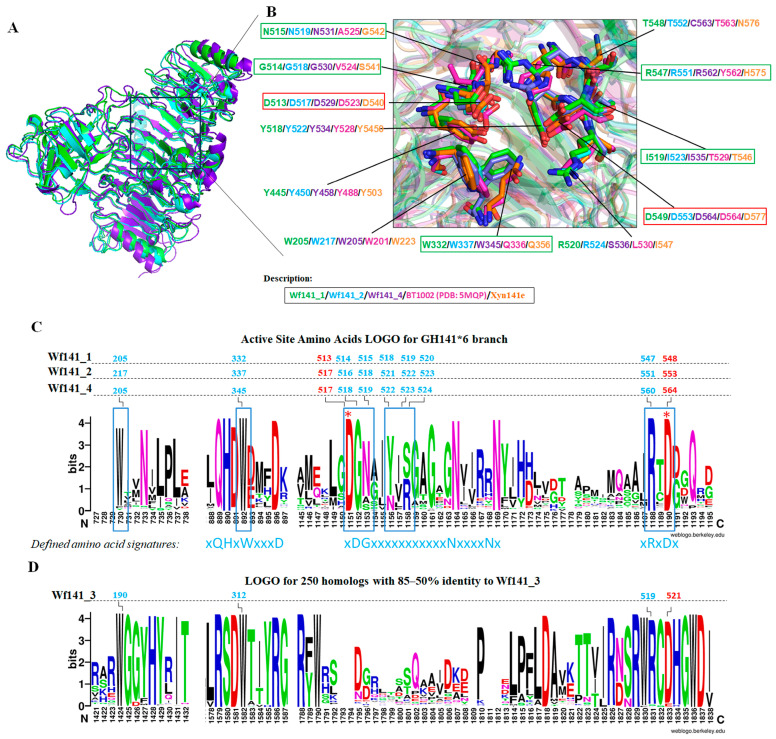
Structural analysis of 3D models of Wf141_1, Wf141_2, Wf141_3, and Wf141_4, as well as previously characterized α-L-fucosidase BT1002 (PDB 5MQP) and endo-1→4-β-D-xylanase Xyn141E of the GH141 family. (**A**) Structural alignment of the AlphaFold predicted 3D models of Wf141_1, Wf141_2, and Wf141_4. (**B**) Structural alignment of the relevant part of the putative active sites of Wf141_1, Wf141_2, Wf141_4, BT1002, and Xyn141E showing the location and differences between some key amino acid residues. The red frame marks conservative asparagine residues that act as nucleophiles and acid bases. The green frame indicates amino acid residues that are conserved in Wf141_1, Wf141_2, Wf141_3, and Wf141_4 but differ in BT1002 and Xyn141E. (**C**) Amino acid sequence logo of the relevant portion of the multiple sequence alignment of the GH146*6 branch members. The blue frame indicates amino acid residues located within a 5 Å radius of the active centers Wf141_1, Wf141_2, Wf141_4, BT1002, and Xyn141E, as indicated in (**B**). The positions of the identified key amino acids in the sequences Wf141_1, Wf141_2, and Wf141_4 are indicated above the logo. Identified conserved amino acid signatures characteristic of the GH141*6 branch are indicated below the logo. Catalytic amino acid residues are marked with an asterisk (*). (**D**) Amino acid sequence logo for Wf141_3 homologs. Amino acid residues present in Wf141_3 as well as in Wf141_1, Wf141_2, and Wf141_4 are indicated above the logo.

We analyzed the AlphaFold predicted 3D models of Wf141_1, Wf141_2, Wf141_3, and Wf141_4. As shown, the Wf141_1, Wf141_2, Wf141_3, Wf141_4, α-L-fucosidase BT1002 (PDB entry 5MQP) and endo-1→4-β-D-xylanaseXyn141E share common for the GH141 family parallel β-helix fold (Figure 4 and Figure S2). However, the surface topology and electrostatic potential of the active site cavities in the predicted models differed significantly from those of BT1002 and Xyn141E. The active sites of Wf141_1, Wf141_2, and Wf141_4 show a much more extended cavity compared to α-L-fucosidase BT1002, indicating their preference for adopting a polymeric substrate (Figure S2). Compared to BT1002 and Xyn141E, the electrostatic potential of the active site surfaces of Wf141s showed to be largely electropositive, indicating their ability to bind electronegative substrates.

A comparative structural analysis was performed on the amino acid residues within the putative active sites of three-dimensional models of Wf141_1, Wf141_2, and Wf141_4, alongside the previously characterized α-L-fucosidase BT_1002 (PDB entry 5MQP) and the endo-1→4-β-D-xylanase Xyn141E (Figure 4A,B). The conserved residues D523 and D564 have previously been shown to be essential for the activity of α-L-fucosidase BT_1002 [23]. Their mutual arrangement in the predicted Wf141_1, Wf141_2, and Wf141_4 models suggests that they are likely to act as an acid/base and nucleophile pair during the hydrolysis of glycosidic bonds (Figure 4B). The composition and arrangement of some amino acids in active sites of Wf141_1, Wf141_2, and Wf141_4, as well as of BT_1002 and Xyn141E, revealed their significant differences. The active sites of Wf141_1, Wf141_2, and Wf141_4 contain a number of polar residues such as Asn (N515, N519, N531 in Wf141_1, Wf141_2, and Wf141_4) and Arg (R547, R551, and R562 in Wf141_1, Wf141_2, and Wf141_4). These residues correspond to non-polar Ala, Gly, heterocyclic His, or aromatic Tyr in the active sites of BT_1002 and Xyn141E. The Gln residue (Q336 in BT_1002 and Q356 in Xyn141E) in the active sites of BT_1002 and Xyn141E corresponds to an aromatic Trp residue in Wf141_1, Wf141_2, and Wf141_4 (W332, W337, or W345). Such aromatic amino acids, often involved in the recognition and binding of certain carbohydrate residues through CH/π interactions [34]. The identified amino acid residues are presumed to facilitate substrate binding and optimal accommodation of sulfated fucosyl residues. It is important to note that these findings are based on computational predictions and necessitate further confirmation via X-ray diffraction studies.

Analysis of the amino acid sequence regions corresponding to the active site residues of Wf141_1, Wf141_2, and Wf141_4 in the multiple alignment of 202 representatives of the GH141*6 branch allowed the identification of conserved amino acid signatures xHQxWxxxD, xDGxxxxxxxxxxxxxxxxxxxxxxxxNxxxxNx, and xRxDx (Figure 4C). Presumably, such amino acid signatures could be characteristic of the fucoidan-active enzymes of the GH141*6 branch of the GH141 family.

Despite the commonality for the GH141 family protein fold of Wf141_3, its active site differs significantly from those of Wf141_1, Wf141_2, and Wf141_4, BT_1002, as well as Xyn141E (Figure S2). The amino acid sequence of Wf141_3 is currently not present in the CUPP database. Analysis of the Wf141_3 amino acid sequence using the CUPP algorithm confirmed that it belongs to the GH141 family, but did not match any similarities to existing subfamilies. Apparently, the Wf141_3 represents a new CUPP branch within the GH141 family. Multiple alignments of the 250 amino acid sequences with 85 to 50% identity to Wf141_3 revealed characteristic patterns for this branch. The identified amino acid pattern of the Wf141_3 cluster was shown to be significantly different from that of the GH141*6 branch (Figure 4C,D).

### 2.2. Expression and Purification of Wf141s

In order to determine the functions of the Wf141s enzymes, the genes of all four enzymes were cloned and produced in *E. coli* ArcticExpress (DE3) cells. A single purification by Ni^2+^-affinity chromatography did not result in pure proteins. According to SDS-PAGE analysis, the enzyme preparations obtained contained multiple minor protein bands (Figure S3). The molecular weights of the major bands of Wf141_1, Wf141_2, Wf141_3, and Wf141_4 preparations corresponded to those predicted and were 72, 77, 74, and 74 kDa, respectively. Wf141_1 and Wf141_2 were further purified by gel-permeation and anion exchange chromatography, respectively, to obtain homogeneous proteins. These two enzymes were further used for a comparative study of their biochemical properties.

### 2.3. Biochemical Properties of Wf141(1–2)

Before studying the biochemical properties of the target enzymes, we screened their functional activity against various substrates, such as 4-nitrophenyl glycosides, fucoidans, and sulfated oligosaccharides (more details in Substrate Specificity, Section 2.4). It was found that all Wf141(1–4) enzymes degrade fucoidans with the formation of sulfated oligosaccharides (Supplementary Materials, Figure S4). Therefore, the C-PAGE assay and fucoidan ShF isolated from the brown alga *Sargassum horneri* were used for further enzyme activity testing.

Metal ions can significantly modulate the activities of enzymes as their cofactors and/or inhibitors. Some of the previously studied endo-fucosidases of the GH107 exhibited catalytic activity only in the presence of bivalent cations [21]. Therefore, the impact of various metal cations on the catalytic activity of the enzymes was evaluated. It was shown that both enzymes Wf141_1 and Wf141_2 are able to cleave ShF fucoidan without the addition of metal ions to the reaction mixture (Figure S5). The ions of Ca^2+^, Mg^2+^, and Ba^2+^ at a concentration of 5 mM had no effect on the catalytic activity of both enzymes. The activity of Wf141_1 was significantly enhanced by K^+^ cations at a concentration of 100 mM. The cations of Na^+^ increase the activity of Wf141_2. The activity of both enzymes was completely inhibited by the presence of 5 mM of Fe^3+^ and Cu^2+^ cations.

The pH and temperature optima were found to be different for Wf141_1 and Wf141_2. Fucanase Wf141_1 was found to be most active in the weakly acidic pH range of 6.0–6.4, while Wf141_2 is active in the neutral pH range of 6.9–7.4 (Figure S5). Fucanase Wf141_1 exhibits maximum activity at a temperature range of 30–45 °C, while Wf141_2 was most active at the low temperature of 20 °C. This is in marked contrast to the temperature optimum for the previously characterized GH141 family endo-xylonase Xyn141E from the thermophilic bacterium *A. thermocellus* (basionim *C. thermocellum*), which is 67–75 °C [25].

Both enzymes lost activity when incubated at temperatures above 45 °C for 30 min. The activity of Wf141_1 was detected after incubation for 24 h in buffers with a pH range of 5.4–8.0 (Figure S5). The Wf141_2 was found to be more tolerant of pH changes. Its activity was detected in the pH range of 4.8 to 9.0. Analysis of the melting temperature (T_m_) of the Wf141_1 and Wf141_2 at buffers with different pH values using differential scanning fluorimetry (DSF) assay revealed pH stability of their folds. It was found that Wf141_1 is most stable in buffers with a pH of 5.4, while for Wf141_2, a pH of 6.0 (Figure S6).

### 2.4. Substrate Specificity of the Wf141(1–2)

The functional role of enzymes of the GH141 family in the degradation of fucoidans has not been described experimentally. The presence of α-L-fucosidase activity in the previously characterized member of the GH141 family, BT1002, may indicate a similar activity among fucoidan-active enzymes of this family. However, the discovery of the endo-xylanase Xyl141E suggests that the enzymes of this family may have different modes of action against glycan molecules. Thus, the GH141 family enzymes can act in either exo- or endo-mode.

In order to determine the mode of action and specificity of the Wf141s, we tested their activity against substrates of different types, such as pNp-glycosides, sulfated fuco-oligosaccharides, and fucoidans of different structures, as well as their derivatives.

#### 2.4.1. Effect of Wf141(1–2) on pNp-Glycosides

In order to evaluate the ability of the enzymes to cleave L-fucose residues or other monosaccharides from non-carbohydrate aglycones, we tested their activity against pNP-glycosides of different structures, such as pNp-α-L-Fucp, pNp-α-L-Glcp, pNp-α-L-Manp, pNP-β-D-Glcp, pNp-α-D-Galp, pNp-β-D-Galp, and pNp-β-D-GlcpNAc. It was found that neither enzyme acted on the pNP-glycosides used, even after a prolonged incubation period (for 48 h). Thus, the enzymes Wf141_1 and Wf141_2 do not possess exo-type phenyl α-L-fucosidase or other glycosidase activity.

#### 2.4.2. Effect of the Wf141(1–2) Against Fucoidans Isolated from Different Brown Algae

**Figure 5:**
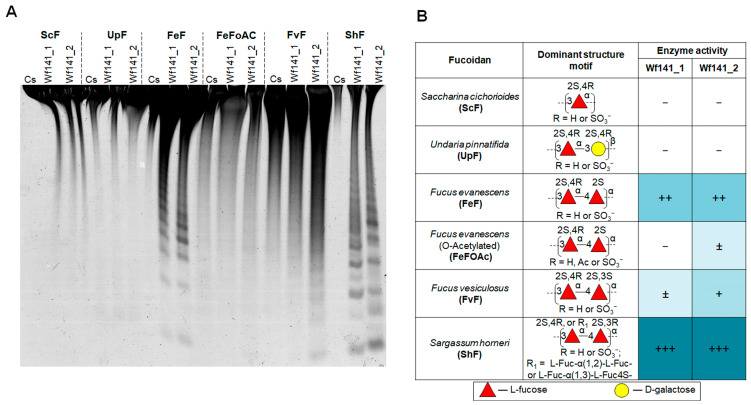
The effect of Wf141_1 and Wf141_2 on fucoidans isolated from the different species of brown algae. (**A**) C-PAGE analysis of the activity of Wf141_1 and Wf141_2 on various fucoidans. Cs—the non-hydrolyzed sulfated polysaccharide. (**B**) The table shows the effects of Wf141_1 and Wf141_2 on fucoidans isolated from different species of brown algae with different structures. The enzymatic activity data were derived from C-PAGE analysis; endo-fucanase activity was not detected (−); barely detectable activity is indicated by (±); low, medium, and high activities are indicated by (+), (++), and (+++), respectively. Carbohydrate residues are depicted according to the symbol nomenclature for glycans as outlined in [39]. Additional repeats of this experiment are shown in Figure S7.

We investigated the endo-fucanase activity of Wf141(1–2) against fucoidans isolated from different brown algae species such as *S. cichorioides* (ScF), *U. pinnatifida* (UpF), *F. evanescens* (FeF), *F. vesiculosus* (FvF), and *S. horneri* (ShF). The fucoidans used in this experiment have different structures (Figure 5B), which allowed us to identify the selectivity of the target enzymes for some structural types of fucoidans. The activity was monitored by the C-PAGE method (Figure 5A).

C-PAGE analysis showed that Wf141_1 and Wf141_2 were unable to hydrolyze fucoidan ScF from *S. cichorioides*, consisting of the sulfated L-fucose residues linked with α-1→3-glycosidic bonds [35]. Fucoidan (galactofucan) UpF isolated from *U. pinnatifida* was also resistant to cleavage with both enzymes. The Wf141_1 and Wf141_2 were shown to be active against fucoidans FeF, FvF, and ShF, backbones of which are composed of alternating α-1→4 and α-1→3-linked residues of sulfated L-fucose [36,37,38]. The formation of a large number of oligosaccharides during fucoidan cleavage by both enzymes indicates their endo-mode of action. This is also consistent with the bioinformatics analysis data on the extended size of the active site cavities of the Wf141_1 and Wf141_2 (Figure S2).

Both enzymes were most active against ShF fucoidan from *S. horneri* composed of 2- and/or 2,3-di-sulfated L-fucose residues (Figure 5A,B). Deacetylated fucoidan FeF from *F. evanescens* with the same repeating linkage type, but other sulfate group positions were less effectively cleaved by the enzymes than ShF. Acetylated fucoidans FeFoAc and FvF were degraded by the enzymes to a lesser extent compared to deacetylated FeF. The data obtained indicate that the type of glycosidic linkages and the position of substituents, such as sulfates and acetates, on L-fucose residues are of great importance for the activity of Wf141_1 and Wf141_2.

#### 2.4.3. Effect of Wf141_2 on the Enzymatically Modified Fucoidans

**Figure 6:**
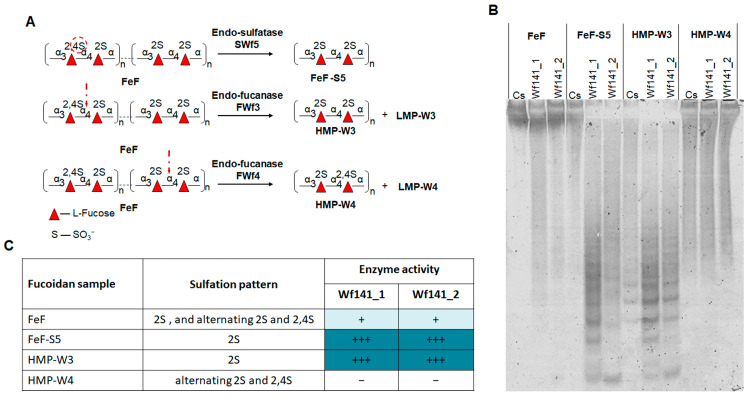
Comparative analysis of the effect of Wf141_1 and Wf141_2 on fucoidan FeF and its derivatives with the regular sulfation. (**A**) Scheme of the preparation of derivatives HMP-W3, HMP-W4, and FeF-S5 from fucoidan FeF using endo-fucanases FWf3, FWf4, and endo-4-*O*-sulfatase SWF5. The red circle indicates the 4-sulfate group targeted by the endo-sulfatase SWf5. The red arrow shows the cleavage sites in fucoidans that are targeted by the endo-fucanases FWf3 and FWf4. (**B**) C-PAGE analysis of the effect of the Wf141_1 and Wf141_2 on fucoidan FeF and its derivatives HMP-W3, HMP-W4, and FeF-S5. Cs—enzyme-untreated sample. (**C**) Table of the effect of Wf141_1 and Wf141_2 on FeF, FeF_S5, HMP-W3, and HMP-W4. (–)—no activity; (+)—low activity; and (+++)—high activity.

To obtain more information on the specificity of Wf141_1 and Wf141_2 towards a certain sulfation pattern in fucoidans, the different enzymatic derivatives of fucoidan FeF were obtained. The backbone of FeF was previously shown to consist of the extended regions [→3-α-L-Fucp2S-1→4-α-L-Fucp2S-1→]_n_ and [→3-α-L-Fucp2,4S-1→4-α-L-Fucp2S-1→]_n_ with mostly regular sulfation [40]. We modified FeF with the previously characterized endo-1→4-α-L-fucanases FWf3 and FWf4 of the GH107 family, as well as the endo-4-*O*-sulfatase SWF5 (subfamily S1_22) to obtain fucoidan derivatives with regular sulfation [16,26]. As a result, the regular 2-sulfated derivatives FeF-S5 and HMP-W3, as well as the derivative HMP-W4 enriched in 2,4-di-sulfation, were obtained. The scheme for obtaining these derivatives is shown in Figure 6A.

The difference in enzyme activity of Wf141_1 and Wf141_2 towards FeF and enzymatically modified FeF (FeF_S5, HMP-W3, HMP-W4) was analyzed by C-PAGE (Figure 6B). As shown, the 2O-sulfated derivatives FeF_S5 and HMP-W3 were cleaved much more efficiently by fucanases Wf141_1 and Wf141_2 compared to native FeF (Figure 6B,C). The opposite behavior of Wf141_1 and Wf141_2 was observed against the 2,4-di-sulfated derivative HMP-W4. This derivative was almost resistant to cleavage by both enzymes. Thus, the 2,4-sulfation negatively affects the ability of Wf141_1 and Wf141_2 to cleave glycosidic bonds in fucoidan molecules. Previously, it was shown that some endo-fucanases of the GH107 and GH168 families were similarly unable to cleave derivatives enriched in 2,4-di-sulfation [13,26,40]. The results obtained demonstrate that the fucoidan-active enzymes of the GH141 family may share common features in selectivity against certain sulfation, as well as endo-fucanases of the GH107 and GH168 families.

#### 2.4.4. Preparation and Structural Analysis of the Reaction Products Produced by Wf141_1 and Wf141_2

**Figure 7:**
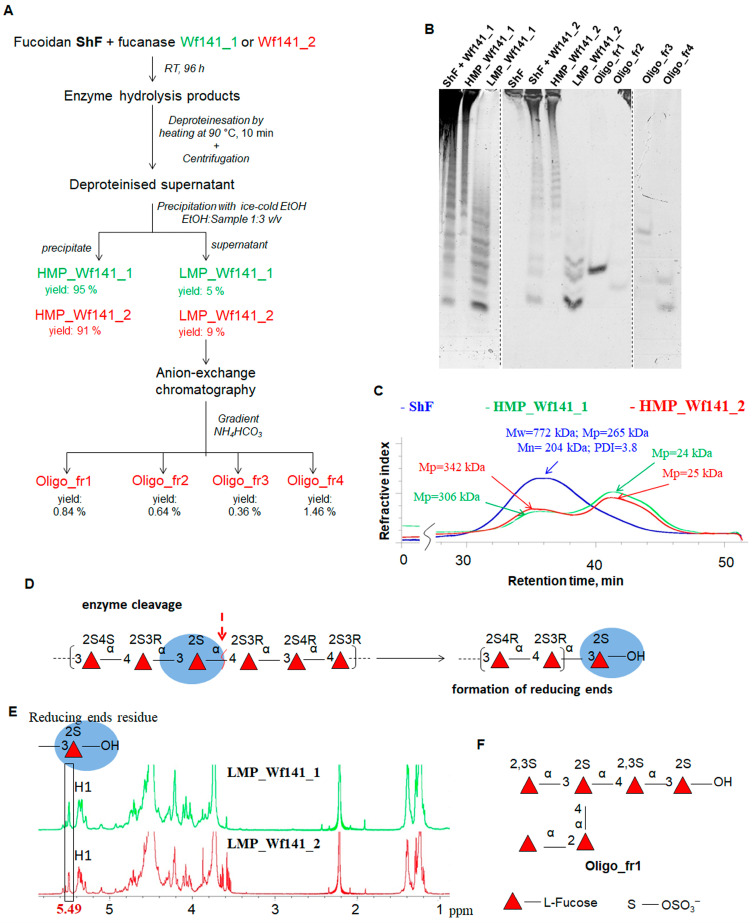
(**A**) Schematic representation showing the separation of reaction products produced with endo-fucanases Wf141_1 and Wf141_2 during hydrolysis of fucoidan ShF. (**B**) C-PAGE analysis of the enzymatic reaction products produced with the Wf141_1 (LMP_Wf141_1 and HMP_Wf141_1) and the Wf141_2 (LMP_Wf141_2, HMP_Wf141_2, Oligo_fr1, Oligo_fr2, Oligo_fr3, and Oligo_fr4). (**C**) SEC of the ShF fucoidan and enzymatic hydrolysis products of HMP_Wf141_1 and HMP_Wf141_2. (**D**) Schematic of enzymatic cleavage of α-1→4-glycosidic linkages showing the formation of the 3-linked Fucp2S reducing end residues (blue oval). The red arrow shows the cleavage sites in fucoidans that are targeted by the endo-fucanases Wf141_1 and Wf141_2. (**E**) ^1^H NMR spectra of the LMP_Wf141_1 and LMP_Wf141_2. The signal at 5.49 ppm corresponding to H1 of the reducing end α-1,3-linked L-fucose residues in LMP fractions is highlighted with a black frame. (**F**) Structure of the oligosaccharide fraction Oligo_fr1.

Structural analysis of hydrolysis products yields comprehensive information regarding enzyme specificity. Accordingly, fucoidan ShF from *S. horneri* underwent complete enzymatic degradation using Wf141_1 and Wf141_2 during an extended 96 h incubation. It was found that fucoidan was not completely hydrolyzed to short oligosaccharides by either Wf141_1 nor Wf141_2. In addition to sulfated oligosaccharides, higher-molecular-weight fucoidan fragments were present in the reaction mixture after cleavage with the enzymes (Figure 7B,C). The high-molecular-weight reaction products (HMP) were separated from the low-molecular-weight (LMP) ones by precipitation with 75% ice-cold ethanol (Figure 7A). The yields of HMP and LMP were approximately 94% and 5% for the Wf141_1, and 90% and 9% for the Wf141_2, respectively. Previously, it was reported that after treatment of fucoidan ShF with endo-fucanase FFA1 of the GH107 family, the yields were 52 and 48%, respectively [41]. Therefore, Wf141_1 and Wf141_2 of the GH141 family demonstrated a more limited effect on the fucoidan ShF compared to the previously described enzymes of the GH107 family. This low LMP yield is probably due to the small number of glycosidic linkages in fucoidan ShF that can be cleaved by Wf141_1 and Wf141_2.

Information on the structural characteristics of the HMP and LMP fractions was obtained via ^1^H and ^13^C NMR spectroscopy. The ^1^H NMR spectra of the HMP fractions did not show discrete signals, indicating their irregular complex structure. The ^1^H NMR spectra of LMP-Wf141_1 and LMP-Wf141_2 fractions were similar to each other, reflecting the structural similarity. Signals at 5.49 ppm were identified in the ^1^H spectra of both LMP fractions (Figure 7E). As shown previously, such signals correspond to the H1 protons of the reducing end 3-linked 2-sulfated residues of L-fucose [38,41]. The formation of terminal 3-linked residues of L-fucose in oligosaccharides produced by enzymatic hydrolysis indicates that both enzymes cleave α-1→4-glycosidic linkages between sulfated L-fucose residues in fucoidan ShF (Figure 7D).

The oligosaccharides of the LMP-Wf141_2 fraction were further separated by anion-exchange chromatography (Figure 7A). Several fractions, Oligo_fr1, Oligo_fr2, Oligo_fr3, and Oligo_fr4, containing the sulfated oligosaccharides were obtained (Figure 7B). The oligosaccharide Oligo_fr1 was of sufficient purity and quantity for its further structural analysis using NMR spectroscopy. The structure of the Oligo_fr1 fraction was determined by 1D and 2D NMR techniques (^1^H, ^13^C, COSY, HSQC, and HMBC) (More data are provided in the Supplementary Materials (Table S1, Figures S8–S10)). Analysis of the Oligo_fr1 spectra showed that this fraction contains a branched sulfated fuco-hexasaccharide (Figure 7F). An oligosaccharide of the same structure was isolated earlier after degradation of fucoidan ShF with the endo-1→4-α-L-fucanase FFA1 [41]. The isolated oligosaccharide was found to contain a 2,3-di-sulfated α-1→3-linked L-fucose residue at the non-reducing end. This confirms that Wf141_2 catalyzes the hydrolysis of the α-1→4-glycosidic linkages between the 2- and 2,3-di-sulfated L-fucose residues in fucoidan ShF.

#### 2.4.5. Effect of Wf141_1 and Wf141_2 on Sulfated Fucooligosacchrides

**Figure 8:**
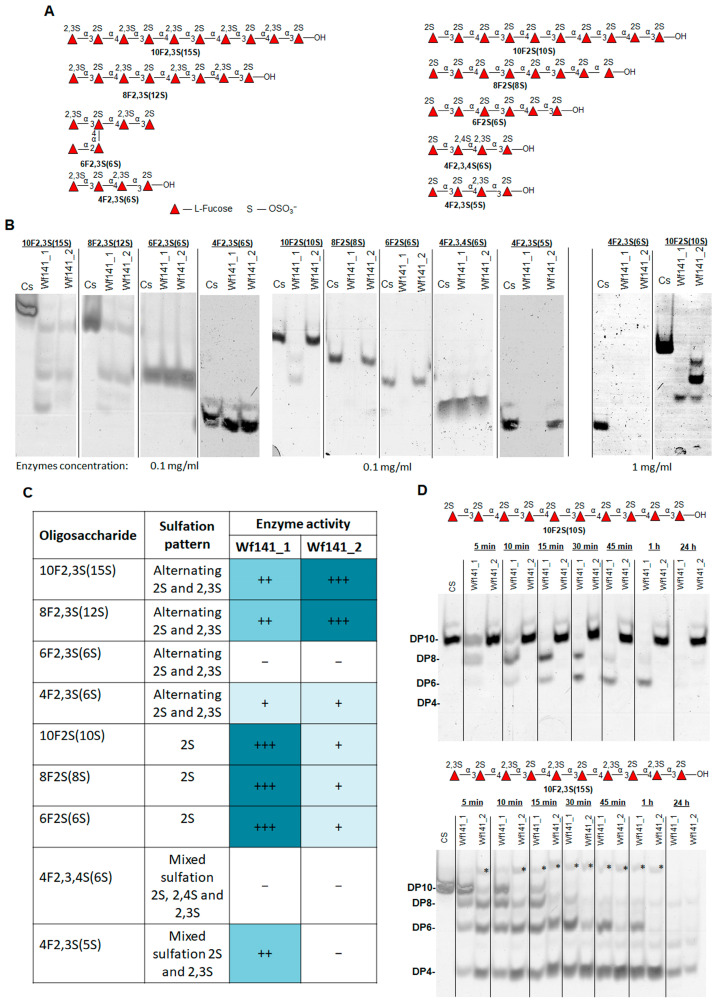
Effect of Wf141_1 and WF141_2 on sulfated fucooligosaccharides with the defined structures. (**A**) Structures of the oligosaccharides 10F2,3S(15S), 8F2,3S(12S), 6F2,3S(6S), 4F2,3S(6S), 4F2,3S(5S), 4F2,3,4S(7S), 10F2S(10S), 8F2S(8S), and 6F2S(6S) used in the experiment. (**B**) C-PAGE analysis of the effects of Wf141_1 and Wf141_2 on sulfated oligosaccharides. Cs—oligosaccharide without treatment with Wf141_1 or WF141_2. (**C**) Table summarizing the effect of the Wf141_1 and Wf141_2 of sulfated oligosaccharides. (−)—no activity; (+)—low or negligible activity; (++)—medium activity; and (+++)—high activity. (**D**) Time course cleavage of the decasaccharides 10F2S(10S) and 10F2,3S(15S) by Wf141_1 and Wf141_2. *—formation of reaction product with higher Mw than native 10F2,3S(15S). Cs—oligosaccharide without treatment with Wf141_1 or WF141_2.

Oligosaccharides with defined structures are the most convenient substrates for detailed characterization of the specificity of glycoside hydrolases. To determine in detail the substrate specificity and the topologies of the sugar-binding subsites of Wf141_1 and Wf141_2, the fucooligosaccharides 10F2,3S(15S), 8F2,3S(12S), 6F2,3S(6S), 4F2,3S(6S), 4F2,3S(5S), 4F2,3,4S(7S), 10F2S(10S), 8F2S(8S), and 6F2S(6S) with defined structures were used as substrates (Figure 8A,B). These oligosaccharides were prepared in-house using different endo-fucanases of the GH107 family [21,41]; some oligosaccharides were further specifically modified with exo-sulfatases to obtain different sulfation patterns [15]. The activity of the enzymes towards the obtained oligosaccharides was detected by C-PAGE.

It was shown that both Wf141_1 and Wf141_2 efficiently cleave oligosaccharides 10F2,3S(15S) and 8F2,3S(12S) containing alternating 2- and 2,3-di-sulfation. The 2-sulfated oligosaccharides 10F2S(10S), 8F2S(8S), and 6F2S(6S) were efficiently degraded by the fucanase Wf141_1, whereas Wf141_2 had a negligible effect (Figure 8B,C). A tenfold increase in the concentration of Wf141_2 resulted in more efficient degradation of the 2-sulfated decasaccharide 10F2S(10S). The tetrasaccharide 4F2,3S(5S), with mixed 2- and 2,3-di-sulfation, was cleaved by the fucanase Wf141_1 but not by Wf141_2. Neither Wf141_1 nor Wf141_2 can cleave the oligosaccharide 4F2,3,4S(6S) with additional 4-sulfation. The data obtained indicate that the specificity of the Wf141_1and Wf141_2 has differences depending on the position of the sulfate groups. Although both enzymes were able to cleave oligosaccharides with 2-sulfation as well as with alternating 2-and 2,3-di-sulfation, the cleavage efficiency was different. This is confirmed by the analysis of the cleavage rates of the 2- and 2,3-di-sulfated decasaccharides 10F2S(10S) and 10F2,3S(15S) (Figure 8C,D). Thus, Wf141_1 preferentially cleaves sites with 2-sulfation, whereas Wf141_2 cleaves sites with alternating 2- and 2,3-di-sulfation.

It is noteworthy that during the enzymatic cleavage of the decasaccharide 10F2,3S(15S), in addition to the low molecular weight oligosaccharides with DP of 8, 6, and 4, a product with a higher molecular weight than the original decasaccharide was formed (Figure 8D, asterisk). This can occur as a result of the transglycosylation reaction. The transglycosylation is usually a side reaction for enzymes that act via the double-displacement mechanism during the hydrolysis of glycosidic linkages [42], suggesting a possible retaining mechanism for the GH141 family of enzymes.

**Figure 9:**
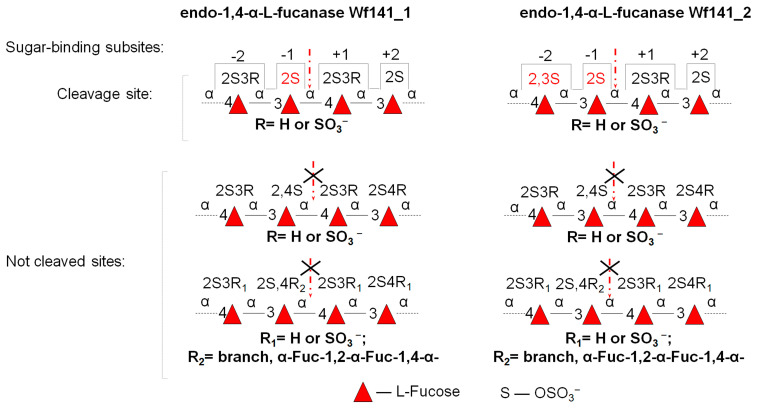
Topology of the sugar-binding subsites of the Wf141_1 and Wf141_2, and selectivity towards specific fucoidan fragments. The arrow indicates the cleavage site in fucoidans. The patterns of sulfation recognized by the enzyme in the carbohydrate-binding subsite are highlighted in red. The numbering of the sugar-binding subsites follows the nomenclature [43].

The degree of polymerization of the oligosaccharides is of great importance for the hydrolysis of glycosidic linkages catalyzed by Wf141_1 and Wf141_2. The ability of glycoside hydrolases to recognize and accommodate a given number of substrate monomers depends on the topology of the sugar-binding subsites in their active sites [43]. As shown, the enzymes at a concentration of 0.01 mg/mL were able to cleave deca-, octa-, and hexasaccharides, but not the tetrasaccharide 4F2,3S(6S) (Figure 8B). Thus, both enzymes prefer to cleave extended substrates, consistent with their endo mode of action. A tenfold increase in the enzyme concentration resulted in the degradation of the tetrasaccharide 4F2,3S(6S). The ability of the enzymes to cleave the terasaccharide 4F2,3S(6S) indicates the presence of at least four significant sugar-binding subsites in the active sites of Wf141_1 and Wf141_2 with subsite topology −2 and +2 (the numbering of the sugar-binding subsites follows the nomenclature [43]). These four subsites appear to be the minimum required for substrate recognition, while the preference for the more extended oligosaccharides indicates a greater number of subsites. As shown, both enzymes are able to cleave the 2,3-di-sulfated tetrasaccharide 4F2,3S(6S) at high concentrations (Figure 8B). However, its derivative 4F2,3S(5S) without the 3-sulfate group at the non-reducing end was cleaved by fucanase Wf141_1 but not by Wf141_2. It is evident that the presence of 2,3-di-sulfation at the −2 subsite is critical for the accommodation and subsequent cleavage of the short substrates by the Wf141_2 enzyme, whereas such sulfation is not essential for Wf141_1. The inability of both enzymes to cleave 2,4-di-sulfated substrates (Figure 6B,C) indicates that the −1 subsite can strictly accommodate only 2-sulfated L-fucose residues. This is supported by the lack of activity against the branched at C4 oligosaccharide 6F2,3S(6S) (Figure 8B). The deduced specificity of the fucanases Wf141_1 and Wf141_2 for specific sites in fucoidans and the topology of their sugar-binding subsites are shown in Figure 9.

Summarizing the obtained data on the structure of the reaction products, the action of the enzymes on fucoidans and oligosaccharides, Wf141_1 and Wf141_2 can be classified as sulfated (1→3;1→4)-α-L-fucan endo-1→4-α-L-fucanase (EC 3.2.1.212). Despite the high degree of amino acid sequence identity and the ability to cleave α-1→4-glycosidic bonds, these enzymes were shown to recognize different patterns of sulfation in fucoidans. Similar complex specificity, depending on the structural environment adjacent to the cleavable glycosidic bond, has been previously reported for members of GH107 and GH168 [13,26].

## 3. Materials and Methods

### 3.1. Reagents and Substrates

*W. fucanilytica* strain CZ1127^T^ was procured from the Korean Collection for Type Cultures (KCTC 42864, 181 Ipsin-gil, Jeongeup-si, Jeollabuk-do, Republic of Korea).

Recombinant sulfatase enzymes—endo-4-O-sulfatase SWF5, and exo-3-O-sulfatase SWF4—derived from the marine bacterium *W. fucanilytica* CZ1127^T^ were prepared according to previously established protocols [15,16]. Similarly, recombinant endo-fucanases FWf3 and FWf4 from the same bacterial source were obtained following the methodology described in [26].

Macroalgal specimens were collected from multiple geographic locations. *Fucus distichus* subsp. *evanescens* thalli were harvested from the intertidal zone at Kunashir Island, Pacific Coast (August 2017), while *Undaria pinnatifida* and *Saccharina cichorioides* samples originated from Troitsa Bay, Sea of Japan, Russian Far East (July and September 2015, respectively). *Sargassum horneri* was collected from Huiquan Bay, Yellow Sea, Qingdao, China (July 2016). Polysaccharide extraction and purification from these macroalgal sources followed established techniques [44]. A commercial fucoidan from *Fucus vesiculosus* (Sigma-Aldrich, Steinheim, Germany; product number F5631, lot number 028K3779V, product code 1000910763) was employed. Acetate-enriched fucoidan preparations isolated from *F. evanescens* and *U. pinnatifida*, as determined by ^1^H NMR spectroscopy, underwent further deacetylation as described in [45].

The 4-desulfated derivative FeF_S5 was prepared via treatment with endo-4-O-sulfatase SWF5 [16], whereas high-molecular-weight fragments HMP-W3 and HMP-W4 were produced through enzymatic degradation using endo-fucanases FWf3 and FWf4, respectively [26].

Sulfated oligosaccharide standards—4F2S(4S), 4F2,3,4S(6S), 6F2S(6S), 8F2S(8S), and 10F2S(10S)—were produced as described in [21]. Additional oligosaccharides (4F2,3S(6S), 6F2,3S(6S), 8F2,3S(12S), and 10F2,3S(15S)) were prepared following established methods [41]. A 3-desulfated tetrasaccharide variant (4F2,3S(5S)) was generated from the parent tetrasaccharide 4F2,3S(6S) through selective desulfation using exo-3-O-sulfatase SWF4 according to the method described in [26].

### 3.2. General Methods

Isolation and purification of genomic DNA were performed using GenElute Bacterial Genomic DNA Kit (Sigma, St. Louis, MO, USA), according to the manufacturer’s protocol. DNA concentration was determined with a Nano-300 microspectrophotometer (Allsheng, Hangzhou, China).

The concentrations of carbohydrates were determined by the phenol–sulphuric acid method using alpha-L-fucose as a standard [46].

Protein concentration was determined by the Bradford method using bovine serum albumin as a standard [47]. Protein purity and molecular weight were estimated by 12% polyacrylamide gel electrophoresis under desaturating conditions (SDS-PAGE) according to the Laemmli protocol [48]. The Precision Plus Protein Standards molecular weight marker (Bio-Rad, Hercules, CA, USA) with molecular weights ranging from 10 to 250 kDa was used as a standard. Gel images were acquired using a GS-800 densitometer (Bio-Rad, Hercules, CA, USA). QuantityOne 4.6.7 software (Bio-Rad, Hercules, CA, USA) was used to calculate the molecular weights of the proteins in the gel.

### 3.3. Amino Acid Sequence Analysis

The identification of the fucoidan-degrading cluster in the genome of *W. fucanilytica* CZ1127T (GenBank: GCA_001697185.1) and putative fucoidan-degrading enzymes was previously described in [13,15]. The amino acid sequences of Wf141_1 (GenBank: ANW96096.1), Wf141_2 (GenBank: ANW96099.1), Wf141_3 (GenBank: WP_068825880.1), and Wf141_4 (GenBank: ANW97461.1) were manually annotated using the dbCAN3 metaserver [16,30] and the Conserved Unique Peptide Pattern (CUPP) server [30]. To assess whether Wf141_1, Wf141_2, Wf141_3, Wf141_4, and previously characterized GH141 members belong to a specific subfamily, their sequences were analyzed using CUPP (CUPP database, June 2024). The distribution of GH141 family members across CUPP branches was visualized using Cytoscape v3.10.2 [49].

For sequence similarity network (SSN) analysis, the amino acid sequences of the GH141 family enzymes were extracted from the CAZy database (www.cazy.org (accessed on 1 November 2025)). Sequences were then used to generate SSN using the Enzyme Function Initiative–Enzyme Similarity Tool (EFI-EST) (https://efi.igb.illinois.edu/efi-est/, accessed in 1 November 2025) using default settings [50]. The obtained SSN (955 sequences) was further clustered using Cytoscape v3.10.2 software, with an Edge cutoff filter of 53% and Organic Layout processing.

A signal peptide was predicted using SignalP (Technical University of Denmark, Version 3.0, Lyngby, Denmark). Multiple sequence alignments (MSA) of Wf141_1, Wf141_2, Wf141_3, Wf141_4, and the GH141 family members were performed using the MAFFT service [51] and Jalview software (ELIXIR-UK resource, Version 3, Hinxton, Cambridgeshire, UK) [52].

Analysis and visualization of spatial structures Wf141_1 (Uniport and AlphaFold access: A0A1B1Y5P3), Wf141_2 (Uniport and AlphaFold access: A0A1B1Y5S7), Wf141_3 (Uniport and AlphaFold access: A0A1B1Y5P0), Wf141_4 (Uniport and AlphaFold access: A0A1B1Y9K1), Xyl141E (Uniport and AlphaFold access: A3DHG8) predicted by the AlphaFold2 server [53], and the α-L-fucosidase BT1002 (PDB entry: 5MQP) was conducted using PyMOL software (PyMOL Molecular Graphics System, version 1.8, Schrödinger, LLC, New York, NY, USA).

Amino acid signatures were generated using MAFFT MSA of proteins corresponding to the GH141*6 CUPP branch (for Wf141_1, Wf141_2, and Wf141_4) or 50–85% homologues (for Wf141_3) with subsequent selection of regions containing key amino acids of Wf141s active sites. Sequence logos of regions containing key active site amino acid residues of Wf141s in MAFFT MSA were generated using WebLogo [54].

### 3.4. Construction and Cloning of the Expression Vectors

The four constructs containing the gene encoding the Q20-N722 region of Wf141_1, the Q29-K726 region of Wf141_2, the L23-K687 region of Wf141_3, and the D16-N730 region of Wf141_4 were designed to contain the N-terminal polyhistidine tag (6His, vector encoded). The constructs were cloned using a restriction-free (RF) cloning strategy [55] similar to that described previously [13]. The genomic DNA of *W. fucanilytica* CZ1127^T^ was used as a template for amplification of the target genes. pCold-II plasmid was used as an expression vector. Primer design and polymerase chain reaction (PCR) conditions were carried out using a service at http://www.rf-cloning.org/ (accessed on 1 August 2025). The primers used for RF cloning are listed in Table 1.

### 3.5. Expression and Purification of the Recombinant Wf141s

The chemically competent E. coli Arctic Express strain (DE3) cells were transformed with pCold-II/*wf141_1*, pCold-II/*wf141_2*, pCold-II/*wf141_3*, and pCold-II/*wf141_4* plasmids using the heat shock procedure and further cultured on lysogenic broth (LB medium) with ampicillin (final concentration 100 μg/mL) in a shaking incubator at 210 rpm and 31 °C for 16 h. The resulting transgenic cell suspensions were inoculated into 100 mL (1:100 *v*/*v*) Terrific broth supplemented with glucose and lactose (DIAEM, Moscow, Russia) for autoinduction of expression. Recombinant bacterial growth in Terrific broth was carried out at 210 rpm and 31 °C until OD_600_ = 0.6–0.8, then the temperature was lowered to 18 °C and growth was continued until the density of the cultures reached saturation (for 24–48 h).

The resulting transgenic bacterial cells were separated from the liquid broth by centrifugation at 7200× *g* for 15 min at 4 °C. The cells were further resuspended in 0.04 M Tris-HCl buffer, pH 7.2, containing 0.15 M NaCl in a mass ratio of 1:10. The bacterial cells were disrupted by sonication using a Sonopuls HD-2070 ultrasonic homogenizer (Bandelin electronic GmbH & Co. KG, Berlin, Germany) at 4 °C in nine cycles (1.5 min each cycle, with a 1.5 min break between cycles). The cell debris was removed by centrifugation at 10,500× *g* for 20 min at 4 °C, and the supernatant was collected. The resulting cell-free extracts were subjected to Ni^2+^ affinity chromatography on a HisTrapHP cartridge (GE Healthcare, Chicago, IL, USA) equilibrated with 0.04 M Tris-HCl buffer, pH 7.2, containing 0.2 M NaCl and 20 mM imidazole. The His-tagged proteins were eluted using a step gradient of 50 and 300 mM imidazole in 0.04 M Tris-HCl buffer, pH 7.2, with 0.2 M NaCl. The fraction eluted with 300 mM imidazole was collected and desalted on HiTrap Desalting cartridges (5 mL, Cytiva, Washington, DC, USA). The obtained enzyme preparations were stored at −20 °C.

The Wf141_1 was further fractionated by gel-permeation chromatography on a Superdex 75 column (L × W, 100 × 1 cm) (GE Healthcare, Chicago, IL, USA) equilibrated with 0.04 M Tris-HCl buffer pH 7.2 containing 0.15 M NaCl. The fractions, which contained homogeneous proteins according to SDS-PAGE analysis, were pooled, concentrated, and stored at −20 °C.

The Wf141_2 was fractionated by anion exchange chromatography on a DEAE-MacroPrep column (Bio-Rad, Hercules, CA, USA) (L × W, 10 × 1.5 cm) with a linear gradient of 0–1 M NaCl in 0.04 M Na-citric buffer, pH 6.9; fractions containing the target protein were pooled, desalted, concentrated, and stored at −20 °C.

### 3.6. Glycosidase Activity Assay

The ability of Wf141_1, Wf141_2, Wf141_3, and Wf141_4 to catalyze the hydrolysis of glycosidic bonds in 4-nitrophenyl (pNp) glycosides (pNp-α-L-fucopyranoside, pNp-β-L-fucopyranoside, pNp-β-D-glucopyranoside, pNp-β-D-galactopyranoside, pNp-β-D-mannopyranoside, pNp-β-D-glucosaminide-N-acetate, and pNp-β-D-xylopyranoside) was evaluated spectrophotometrically. Reaction mixture (150 μL) containing 0.01 mg/mL enzyme solution and pNp-glycosides solution 0.5 mg/mL in 0.04 M Tris-HCl buffer, pH 7.2, containing 0.15 M NaCl was incubated for 48 h at 30 °C. The reactions were stopped by adding 100 μL of 1 M Na_2_CO_3_ solution, and the pNp produced was quantified spectrophotometrically at 410 nm using a PowerWave XS plate reader (BioTek, Winooski, VT, USA).

### 3.7. Endo-Fucanase Activity Assay

The reaction mixture consisted of 20 μL of an aqueous fucoidan solution (10 mg/mL), along with 20 μL of Wf141_1 or Wf141_2 (0.1–1 mg/mL) in 0.04 M Tris-HCl buffer (pH 7.2), and was incubated for 24 h. The endo-fucanase activity was detected via charged oligosaccharide bands occurance in the C-PAGE gel as described in [15].

### 3.8. Effect of the Wf141(1–2) Against Sulfated Fucooligosaccharides

A reaction mixture consisting of 6 µL of water solution of the fucooligosaccharides 10F2S(10S), 8F2S(8S), 6F2S(6S), 4F2,3,4S(6S), 10F2,3S(15S), 8F2,3S(12S), 6F2,3S(6S), 4F2,3S(6S), or 4F2,3S(5S) (0.5 mg/mL) and 6 µL of the enzyme (0.2 or 2 mg/mL) in 0.04 M Tris-HCl buffer, pH 7.2, was incubated for 18 h. The reaction was stopped by heating at 85 °C for 5 min. The fucanase activity was detected by C-PAGE. The presence of catalytic activity was estimated by changes in electrophoretic mobility or disappearance of reaction products on the electropherogram.

The time course of the cleavage of the sulfated decasaccharides 10F2S(10S) and 10F2,3S(15S) by Wf141_1 and Wf141_2 was studied in a similar manner. Reaction mixtures containing 40 µL of substrate (0.25 mg/mL) and 60 µL (0.1 mg/mL) of enzyme solution in 0.04 M Tris-HCl buffer, pH 7.2, were incubated for 24 h. Aliquots (12 µL) of the reaction mixture were taken after 5, 10, 15, 30, 45, 60 min, and 24 h of incubation with the enzyme. The reaction was then stopped by heating at 85 °C for 5 min. Enzymatic activity was detected by the C-PAGE.

### 3.9. Influence of Multivalent Metal Ions on the Activities of Wf141(1–2)

Reaction mixtures containing 4 µL of ShF water solution (20 mg/mL), 10 µL of 0.04 M Tris-HCl buffer (pH 7.2), 4 µL of enzyme solution (0.1–0.5 mg/mL) and 2 µL of a solution of an appropriate salt (0.1 M of CaCl_2_, BaCl_2_, MgCl_2_, NiSO_4_, MnCl_2_, CuSO_4_, FeCl_3_; 0.2 M of NaCl or KCl) were incubated at 35 °C for 1 h. The reaction was stopped by freezing. Activity levels were determined by C-PAGE as described above.

### 3.10. pH Optimum Determination for the Activities of Wf141(1–2)

Reaction mixtures, containing 4 µL of enzyme (0.1–0.5 mg/mL), 4 µL of ShF water solution (20 mg/mL), 10 µL of buffers with various pH values (0.3 M Na-succinate buffers with pH range of 3.7–6.5; 0.3 M Tris-HCl buffers with pH values from 6.5 to 8.5 or 0.2 M Na-borate buffer, pH 9.0) and 2 µL of 2 M KCl (for Wf141_1 mixture) or 2 µL of 2 M NaCl (for Wf141_2 mixture) solution were incubated for 30 min at 30 °C for Wf141_1 or at 20 °C Wf141_2. The reaction was stopped by freezing. Activity levels were monitored by C-PAGE as described above.

### 3.11. Optimum Temperature Determination for the Activities of Wf141(1–2)

Reaction mixtures, containing 4 µL of enzyme solution (0.1–0.5 mg/mL), 4 µL of ShF solution (20 mg/mL), 10 µL of 0.3 M Na-succinate buffer pH 6.4, and 2 µL of 2 M KCl solution (for Wf141_1), or 10 µL of 0.3 M Tris-HCl buffer pH 7.2 and 2 µL of 2 M NaCl solution (for Wf141_2) were incubated at different temperatures of 4, 8, 15, 20, 25, 30, 37, 45, 55, 60, or 70 °C for 30 min. The reaction was stopped by freezing. Activity levels were monitored by C-PAGE as described above.

### 3.12. pH Stability Determination for the Activities of Wf141(1–2)

Mixtures containing 5 µL of enzyme solution (0.1–0.5 mg/mL) and 5 µL of buffers with different pH values (0.3 M Na-succinate buffer with a pH range of 3.7–6.5; 0.3 M Tris-HCl buffer with a pH range of 6.5 to 8.5 or 0.2 M Na-borate buffer, pH 9. 0) were incubated at 12 °C for 24 h. Then 11 µL of 0.3 M Na-succinate buffer, pH 6.4, supplemented with 3 µL of 2 M KCl solution (for Wf141_1) or 11 µL of 0.3 M Tris-HCl buffer, pH 7.2, supplemented with 3 µL of 2 M NaCl (for Wf141_2) and 6 µL of ShF solution (20 mg/mL) were added. Resulted reaction mixtures were incubated for 30 min. Reaction was stopped by freezing. Activity levels were monitored by C-PAGE as described above.

### 3.13. Temperature Stability Determination for the Activities of Wf141(1–2)

Mixtures containing 5 µL of enzyme (0.1–0.5 mg/mL) and 5 µL of 0.3 M Na-succinate buffer, pH 6.4, (for Wf141_1), or 0.3 M of Tris-HCl buffer, pH 7.2, (for Wf141_2) were incubated at different temperatures (4, 8, 15, 20, 25, 30, 37, 45, 55, 60, and 70 °C) for 30 min. Then, 11 µL of 0.3 M Na-succinate buffer, pH 6.4, supplemented with 3 µL of 2 M KCl (for Wf141_1), or 11 µL of 0.3 M Tris-HCl buffer, pH 7.2, supplemented with 3 µL of 2 M NaCl (for Wf141_2), and 6 µL of ShF solution (20 mg/mL) were added. Reaction mixtures were incubated for 30 min for Wf141_1 and Wf141_2 mixtures, respectively. The reaction was stopped by freezing. Activity levels were monitored by C-PAGE as described above.

### 3.14. Differential Scanning Fluorimetry (DSF)

The DSF was performed according to the main criteria discussed in [38] with minor modifications. Reaction mixtures (60 µL) contained 3 µL of 20× Protein Orange dye 5000× stock solution (Lumiprobe, Moscow, Russia), 11 µL of Wf141_1 or Wf141_2 (2 mg/mL), and 46 µL of buffers with different pH values (0.3 M Na-succinate buffers with pH range from 3.7 to 6.5; 0.3 M Tris-HCl buffers with pH range from 6.5 to 8.5 or 0.2 M Na-borate buffer, pH 9.0). Aliquots of 50 µL of the resulting mixtures were added to a 96-well PCR plate, sealed with an optically clear film (Accumax Lab Devices Pvt. Ltd., Gandhinagar, India), and analyzed using the QuantStudio 5 instrument in the melting curve mode (Thermo Fisher Scientific, Waltham, MA, USA). The melting temperatures (Tm) of the proteins at different pH values were determined using the Origin Lab utility (OriginPro, Version 2021, OriginLab Corporation, Northampton, MA, USA) by fitting the experimental data to the Boltzmann equation.

### 3.15. Preparation and Isolation of Enzymatic Hydrolysis Products

Fucoidan ShF from the brown alga *S. horneri* (500 mg) was dissolved in 49 mL of 0.04 M Tris-HCl buffer, pH 7.2, containing 0.1 M KCl (for Wf141_1) or 0.1 M NaCl (for Wf141_2). To this mixture, 1 mL of Wf141_2 (0.05 mg/mL) solution in the same buffer was added. The reaction was performed at 30 °C (for Wf141_1) or 20 °C (for Wf141_1) for 96 h. After 72 h, 100 µL of Wf141_2 with a concentration of 2.5 mg/mL was added to the reaction mixture and incubated further 24 h. The reaction mixture was then deproteinized by heating to 80 °C for 15 min, followed by centrifugation at 12,000× *g* for 30 min. The high-molecular-weight reaction products (HMP_Wf141_1 or HMP_Wf141_2) were precipitated with ice-cold ethanol 96% at a ratio of 1:3 (*v*/*v*), and the precipitate was separated by centrifugation at 11,000× *g* for 40 min. The supernatant containing the low-molecular-weight reaction products (LMP_Wf141_2 or LMP_Wf141_2) was evaporated under vacuum to remove ethanol and then diluted with distilled water to 10 mL. The fraction LMP_Wf141_2 was subjected to anion-exchange chromatography on a Q-Sepharose HP (1 × 10 cm) column (GE Healthcare, Chicago, IL, USA) equilibrated with water. Oligosaccharides were eluted with a linear gradient of NH_4_HCO_3_ solution from 0 to 2.5 M (120 × 120 mL). The volume of the fractions was 1 mL, and the flow rate was 1 mL/min. The content of carbohydrates in fractions was determined by the phenol–sulfuric acid method. Fractions containing carbohydrates were further analyzed by C-PAGE. Homogeneous oligosaccharide fractions were pooled, desalted by evaporation or HiTrap desalting column (5 mL, Cytiva, Washington, DC, USA), and then lyophilized. The structures of the oligosaccharides obtained were determined by NMR spectroscopy.

### 3.16. Determination of the Molecular Weight of Fucoidan and Its Enzymatic Derivatives

SEC-based analysis of polysaccharide molecular weights was conducted using an Agilent 1100 Series HPLC system (Agilent Technologies, Waldbronn, Germany) outfitted with refractive index detection and dual Shodex OHpak columns (SB-805 HQ and SB-803 HQ; Showa Denko, Tokyo, Japan) in series, as previously described in [13].

### 3.17. NMR Spectroscopy

Nuclear magnetic resonance (NMR) analyses, including one-dimensional (^1^H, ^13^C, TOCSY) and two-dimensional (COSY, ROESY, HMBC, HSQC) experiments, were conducted using Avance III 700 MHz (Bruker BioSpin AG, Fällanden, Switzerland) and Avance II 500 HD (Bruker BioSpin GmbH, Ettlingen, Germany) instruments. For spectral acquisition, 10–20 mg of polysaccharides or 2–4 mg of oligosaccharides were dissolved in 550 µL of D_2_O, with 1 µL of acetone serving as an internal chemical shift reference (δ_H = 2.225 ppm; δ_C = 31.45 ppm). Each solution was placed in a 5 mm NMR tube and allowed to thermally equilibrate inside the spectrometer before measurement. All NMR data were collected at a temperature of 35 °C.

## 4. Conclusions

Glycoside hydrolases of the GH141 family are widespread in both terrestrial and marine microorganisms, indicating their association with enzyme systems involved in the degradation of various polysaccharides of terrestrial and marine origins. The high abundance of genes encoding GH141 family enzymes in fucoidan-degrading marine bacteria may indicate their key role in fucoidan degradation. However, the function of the fucoidan-active enzymes of the GH141 family was hypothetical until now.

The present work describes for the first time the functional characterization of four fucoidan-active glycoside hydrolases, Wf141_1, Wf141_2, Wf141_3, and Wf141_4, of the GH141 family from the fucoidan-degrading cluster of the marine bacterium *W. fucanilytica* CZ1127^T^. It was shown that all four enzymes, Wf141_1, Wf141_2, Wf141_3, and Wf141_4, possess endo-fucanase activity. Detailed analysis of the specificities of Wf141_1 and Wf141_2 shows that these enzymes catalyze the hydrolysis of α-1→4-glycosidic linkages between sulfated L-fucose residues in (1→3;1→4)-α-L-fucoidans. The Wf141_1 and Wf141_2 have been classified as sulfated (1→3;1→4)-α-L-fucan endo-1→4-α-L-fucanases (EC 3.2.1.212). Our results revealed that Wf141_1 and Wf141_2 exhibit selectivity toward distinct sulfation patterns in fucoidan molecules. Endo-fucanase Wf141_1 showed a preference for cleaving fucoidan fragments [→3-α-L-Fucp2S-1→4-α-L-Fucp2S-1→]_n_, whereas endo-fucanase Wf141_2 preferred fragments [→3-α-L-Fucp2S-1→4-α-L-Fucp2,3S-1→]_n_. The ability of Wf141_1 and Wf141_2 to recognize different sulfation patterns is mediated by the different abilities of their sugar-binding subsites in the active sites to adopt L-fucose residues with specific sulfation. Acetylation of L-fucose residues in fucoidans hinders glycosidic bond cleavage by these enzymes. Thus, the specificity of the GH141 family members may depend not only on the nature of the substrate (arabinoxylan, pectin, or fucoidan), the mode of action (exo- or endo-), the type of glycosidic linkages, but also on the specific positions of the substituents on the monosaccharides adjacent to the cleavable glycosidic linkage.

SSN clustering and CUPP analysis of the sequences Wf141_1, Wf141_2, Wf141_3, Wf141_4, and other GH141 family members revealed that Wf141_1, Wf141_2, Wf141_3, and Wf141_4, as well as the previously characterized endo-β-1→4-β-D-xylonase Xyl141E and α-L-fucosidase BT1002, belong to different clusters and branches. SSN-classified clusters 5 and 25, as well as the CUPP branch of GH141*6, may be associated with endo-fucanases of the GH141 family. These data will help researchers to identify fucoidan-active enzymes of the GH141 family and to find new unidentified fucoidan-degrading microorganisms. However, further verification of the functional activities of representatives of the identified clusters is required for confirmation.

Our data showed that fucoidan-active members of the GH141 family, similar to previously characterized endo-fucanases of the GH107, GH168, GH174, and GH187 families, can be involved in the endo-mode depolymerization of fucoidans into short oligosaccharides. This is in contrast to the previously hypothesized role of GH141 family enzymes as α-L-fucosidases in fucoidan degradation. The present data reveal new, previously unknown details of fucoidan degradation by marine bacteria and evidence the novel functional activity within the GH141 family.

## Figures and Tables

**Table 1:** The list of primers used for RF-cloning of the genes encoding the Wf141_1, Wf141_2, Wf141_3, and Wf141_4 from *W. fucanilytica* CZ1127^T^.

Enzyme Name	Primer Sequences, 5′–3′ (F—Forward Primer; R—Reverse Primer)
Wf141_1	F: CCGAGAACCTTTACTTCCAGGGGCAATCAAAGGCAGATTTTTATATAT
	R: TCTTAGATTCTGTGCTTTTAAGCAGAGATTACCTATTAATTTACGACTTTAACCAAACCT
Wf141_2	F: CCGAGAACCTTTACTTCCAGGGGCAAACTGAACAAGTAGCAAAAGCT
	R: TCTTAGATTCTGTGCTTTTAAGCAGAGATTACCTATTACTTCACTTCAAGTAAACCAATCT
Wf141_3	F: CCGAGAACCTTTACTTCCAGGGGTTAGATATTTATGTGAGTCCGAATGGG
	R: TCTTAGATTCTGTGCTTTTAAGCAGAGATTACCTATTTCTTTAGCTCTTCTGAAACACCC
Wf141_4	F: CCGAGAACCTTTACTTCCAGGGGGATTCTAGTAATAAAATTGAATTATATGTTTCTGT
	R: TCTTAGATTCTGTGCTTTTAAGCAGAGATTACCTATTAATTCTTTATAATTCCCATCCTTGA

The underlined and the non-underlined sequences are the vector-specific primers and the gene-specific primers, respectively.

## Data Availability

The original contributions presented in this study are included in the article/Supplementary Materials. Further inquiries can be directed to the corresponding author.

## Supplementary Materials

The following supporting information can be downloaded at: https://www.mdpi.com/article/10.3390/ijms27010443/s1.
