# Supplementary material for: The Discovery of Endo-Fucanases in the GH141 Family: A Novel Functional Activity Within the Family
Source: Int J Mol Sci. 2025 Dec 31;27(1):443. doi: 10.3390/ijms27010443 (PMC12786026; doi:10.3390/ijms27010443)
Supplement: Supplementary file 1 [file ijms-27-00443-s001.zip › ijms-4056016-supplementary.pdf]

Supplementary matherials

The Discovery of Endo-Fucanases in the GH141 Family: A Novel Functional Activity Within the Family

Nikita Konstantinovich Rubtsov [1,†], Artem Sergeevich Silchenko [1,*,†], Marina Petrovna Isaeva [2], Roman Alekseevich Shkrabov [1], Anastasiya Olegovna Zueva [1], Mikhail Igorevich Kusaykin [1] and Svetlana Pavlovna Ermakova [1]

[1] Laboratory of Enzyme Chemistry, G.B. Elyakov Pacific Institute of Bioorganic Chemistry, Far-Eastern Branch of the Russian Academy of Sciences, 159, Prospect 100-Let Vladivostoku, 690022 Vladivostok, Russia; rubtsov.nk@yandex.ru (N.K.R.); shkrabov.ra@outlook.com (R.A.S.); zstasya95@gmail.com (A.O.Z.); mik@piboc.dvo.ru (M.I.K.); svetlana_ermakova@hotmail.com (S.P.E.)

[2] Laboratory of Marine Biochemistry, G.B. Elyakov Pacific Institute of Bioorganic Chemistry, Far-Eastern Branch of the Russian Academy of Sciences, 159, Prospect 100-Let Vladivostoku, 690022 Vladivostok, Russia; issaeva@gmail.com

* Correspondence: artem.silchencko@yandex.ru; Tel.: +7-(423)-231-07-05

† These authors contributed equally to this work.

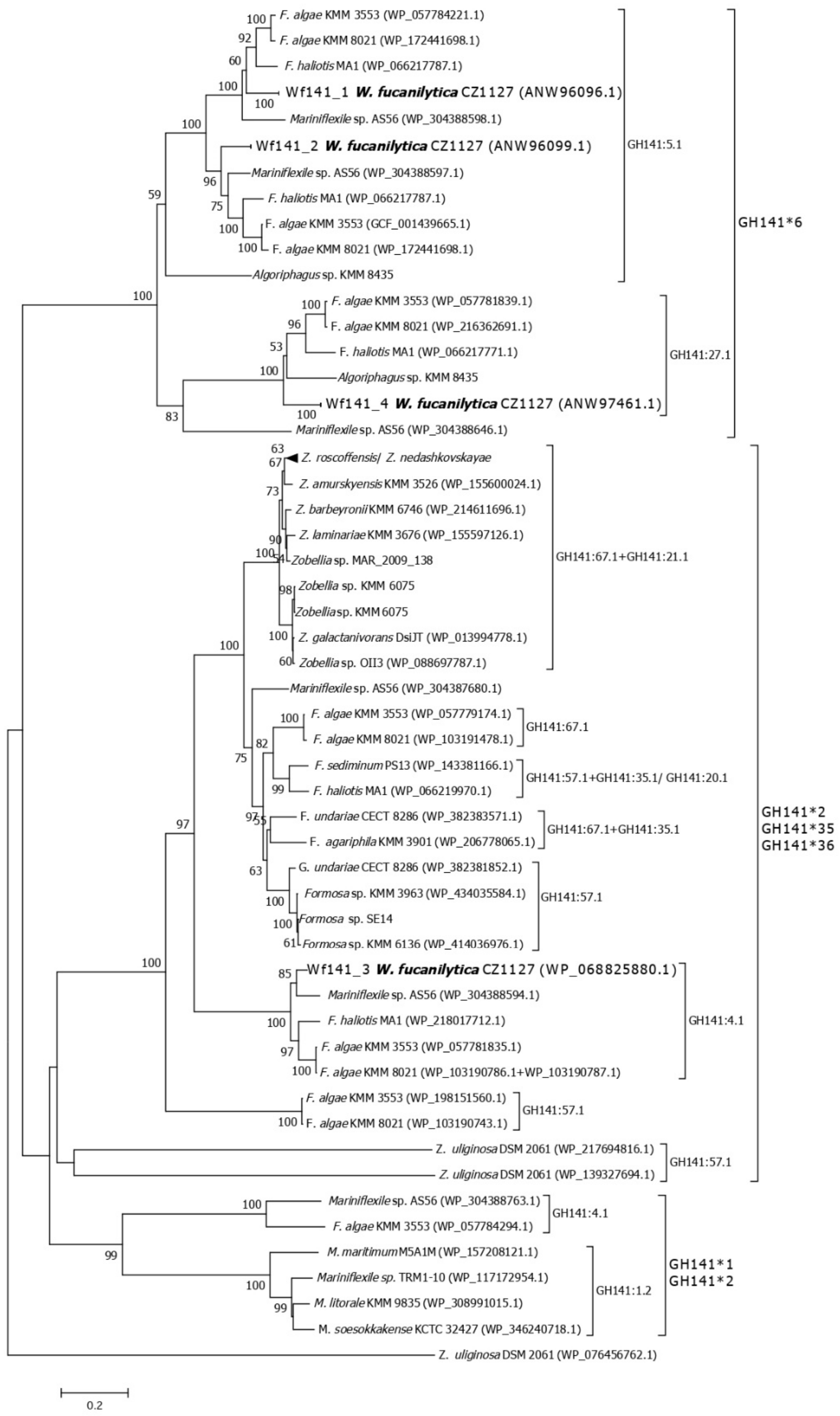

Figure S1. Phylogenetic tree showing the distribution of GH141 family enzymes among certain marine bacteria (*Formosa*, *Algoriphagus*, *Mariniflexile*, *Zobellia* etc.)

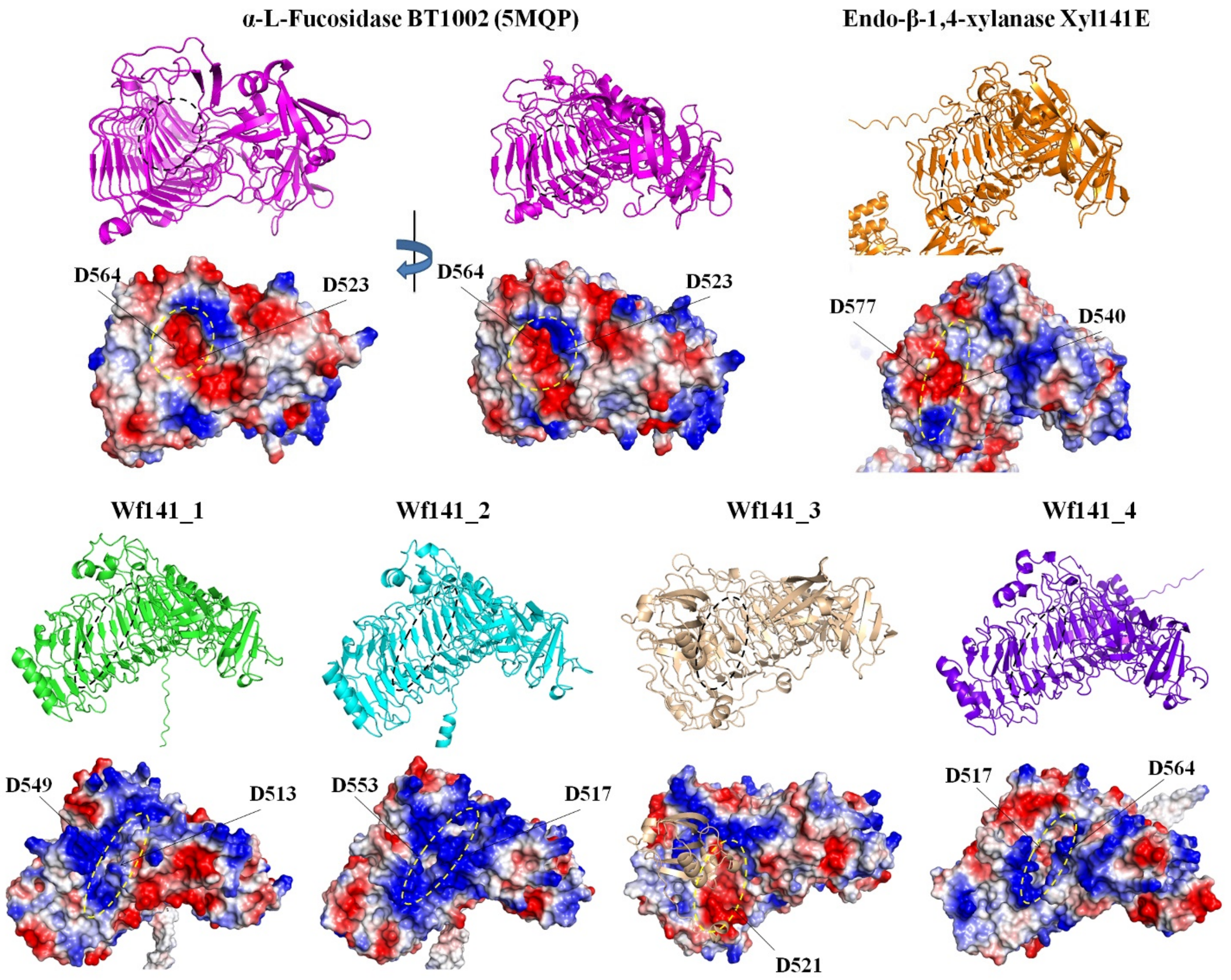

Figure S2. Spatial structures of characterized α-L-fucosidase BT1002 (PDB 5MQP) of the GH141 family, and AlphaFold predicted models of endo-1,4-β-xylanase Xyn141E, Wf141_1, Wf141_2, Wf141_3, Wf141_4. Active sites are shown in yellow dotted line. Asparagine residues (D) acting as nucleophiles and acid-bases in the active sites of the enzymes are indicated by a black arrow.

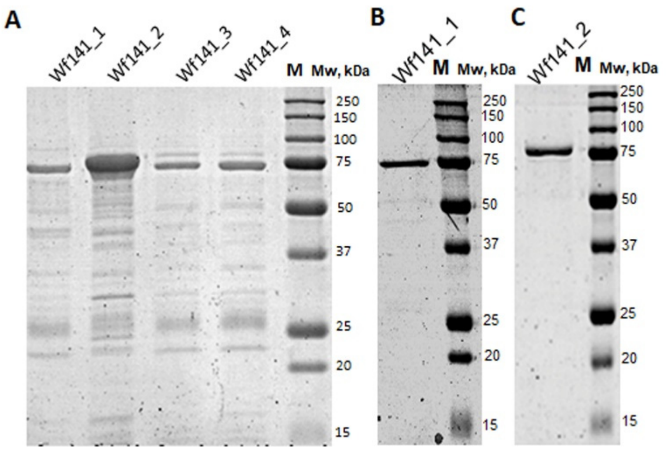

FigureS3. SDS-PAGE of the recombinant Wf141_1, Wf141_2, Wf141_3 and Wf141_4 after Ni-IMAC chromatography (**A**), and Wf141_1 and Wf141_2 further purified by gel-permeation (**B**) and anion exchange chromatography (**C**). M – molecular weight marker (Bio-Rad).

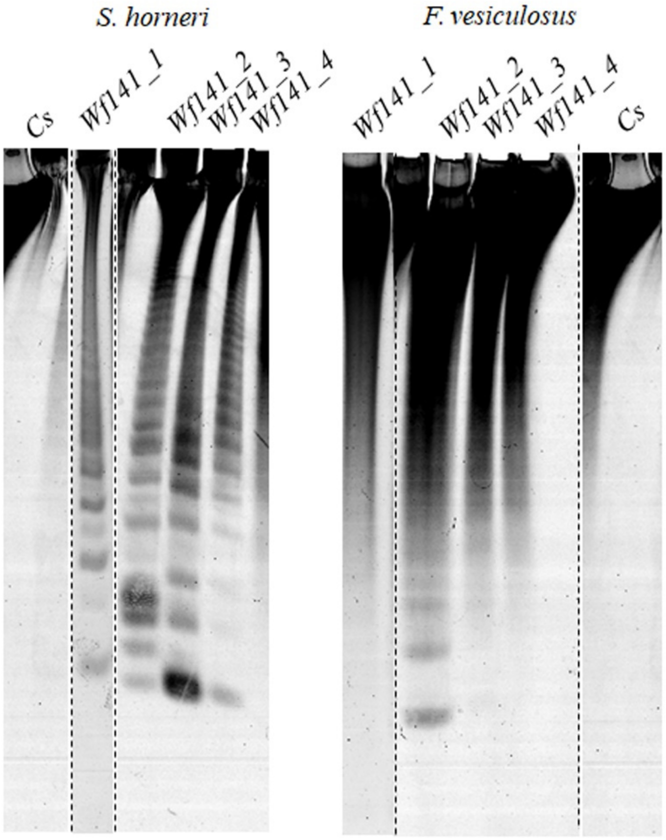

Figure S4. C-PAGE analysis of the activity of Wf141_1, Wf141_2, Wf141_3 and Wf141_4 on fucoidans ShF and FvF isolated from brown algae *S. horneri* and *F. vesiculosus*. Cs – enzyme-untreated sample.

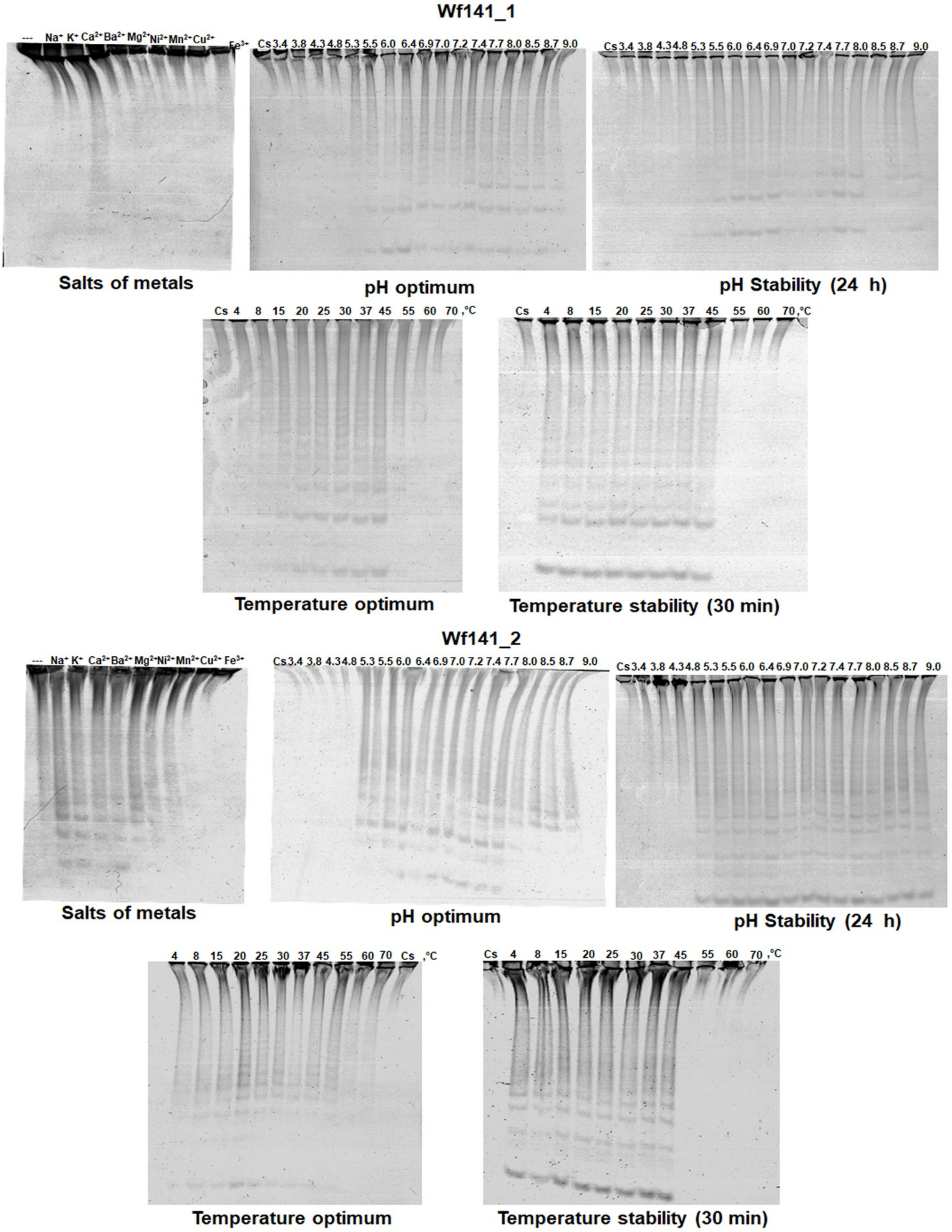

Figure S5. C-PAGE analysis of Wf141_1 and Wf141_2 activity at different pH values, temperatures and in the presence of different metal salts. Cs – enzyme-untreated sample.

Wf141_1

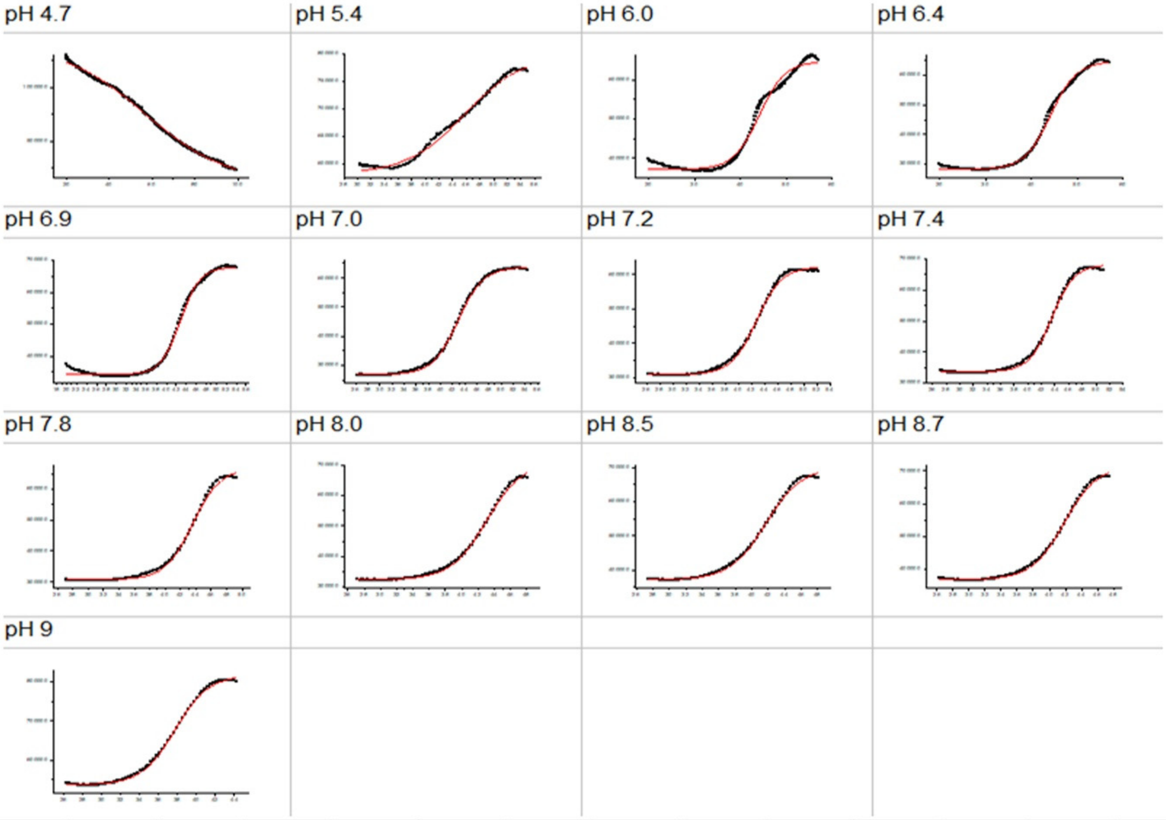

pH	4.7	5.4	6.0	6.4	6.9	7.0	7.2	7.4	7.8	8.0	8.5	8.7	9
Tm	—	45.7	44.1	44.2	42.8	42.9	42.8	43.5	43.6	43.1	41.7	41.9	37.7

Wf141_2

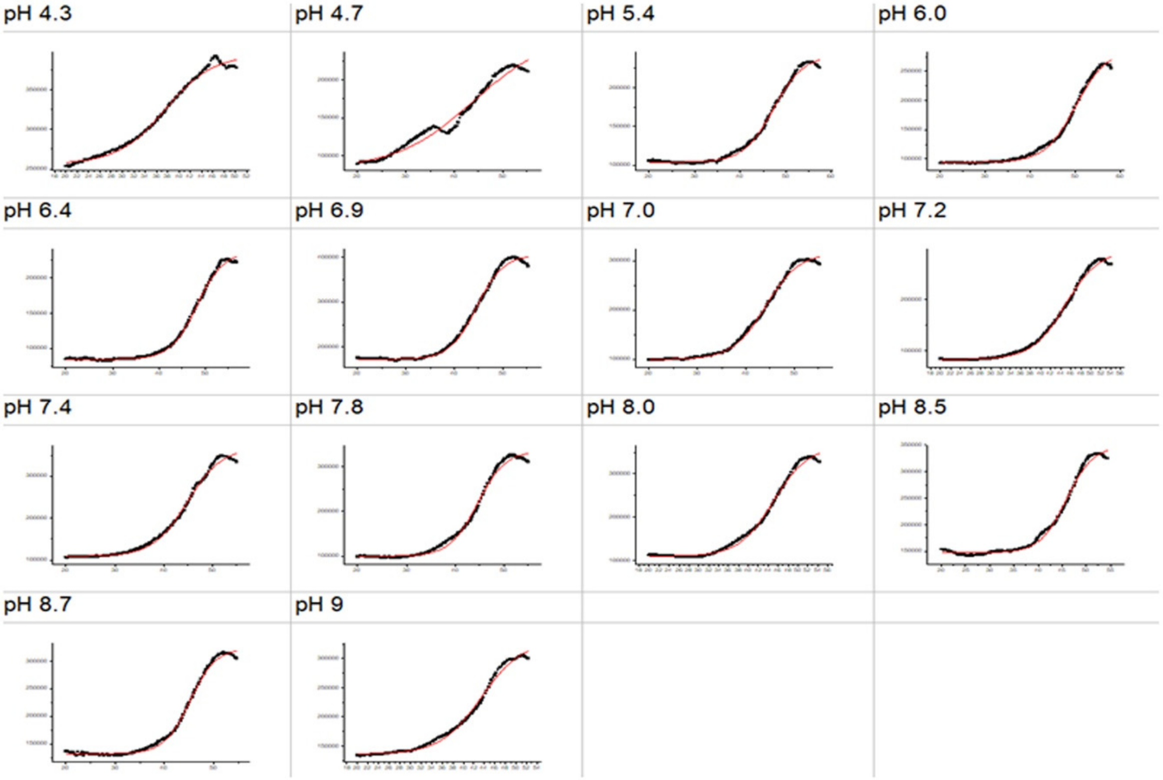

pH	4.3	4.7	5.4	6.0	6.4	6.9	7.0	7.2	7.4	7.8	8.0	8.5	8.7	9
Tm	37.6	44	47	50	48	44.6	44.1	44.9	44.8	44.6	45	45.9	45.2	43.4

Figure S6. Determination of melting temperatures (Tm, ºC) of Wf141_1 and Wf141_2 in buffers with different pH values using the Differential Scanning Fluorimetry (DSF) method.

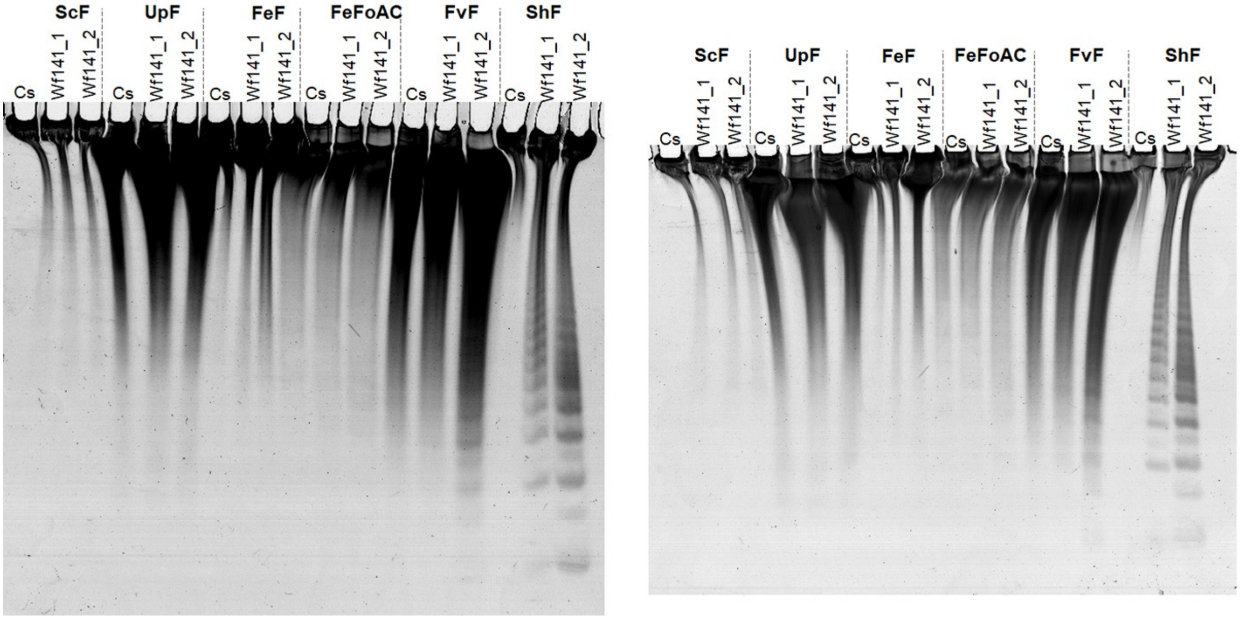

Figure S7. Effect of Wf141_1 and Wf141_2 on fucoidans isolated from different brown algae. This figure presents additional experiments corresponding to those shown in Figure 5A.

Determination of the Oligo_fr1 structure by Nuclear Magnetic Resonance (NMR) spectroscopy.

As shown, the proton spectrum of the Oligo_1 fraction contained 6 anomeric protons (Figure S6). ^{1}H signals were assigned according to the COSY spectrum, which provided spin-spin couplings between neighboring protons. ^{13}C signals were assigned according to the heteronuclear HSQC spectrum, which provided correlations between signals of protons and signals of carbons directly linked to them. The resulting chemical shifts of ^{1}H and ^{13}C of Oligo_1 are grouped in Table S1. The linkage types and their sequences between fucose residues in Oligo_fr1 were deduced from the HMBC spectrum (Figure S7). The HMBC spectrum showed correlation between carbon atoms of residues A_1, C_1, D_1, E_1, F_1 and protons of residues D_4, B_3, C_4, D_3 and A_2, respectively (Figure S6). The position of the sulfate groups in the oligosaccharide was deduced by comparing the chemical shifts of their protons with those of L-fucose (H1 = 5.19, H2 = 3.76, H3 = 3.85, H4 = 3. 80, H5 = 4.19, H6 = 1.20) and the chemical shifts of its carbons with those of α-methyl-L-fucopyranoside (C1 = 100.5, C2 = 69.0, C3 = 70.6, C4 = 72.9, C5 = 67.5, C6 = 16.5). Sulfation at position 2 was inferred from the downfield shift observed for H2 (0.8-0.9 ppm) relative to the L-fucose residue and for C2 (4-6 ppm) relative to α-methyl-L-fucopyranoside. The downfield shift of H3 (0.9-1.0 ppm) and C3 (4-6 ppm) allowed us to infer sulfation at position 3. The data obtained indicate that the Oligo_1 fraction is a branched sulfated fucohexasaccharide with the structure shown in Figure S8.

Table S1. The chemical shifts (ppm) of C and H of the fraction Oligo_fr1.

Residue	Chemical shifts, ppm				
	**H1/C1**	**H2/C2**	**H3/C3**	**H4/C4**	**H5/C5**
α-L-Fuc-(1→2)- (**F**)	5.1	3.84	3.92	3.8	4.3

	100.27	68.7	69.77	72.55	68.47
→2)-α-L-Fuc-(1→4)- (**A**)	5.58	3.74	4.13	3.84	4.32
	97.99	79.3	69.1	68.7	67.6
α-L-Fuc-(2,3SO_3^-)-(1→3)- (**E**)	5.32	4.577	4.83	4.23	4.6
	97.8	73.26	75.84	71.22	66.96
→3,4)-α-L-Fuc-(2SO_3^-)-(1→4)- (**D**)	5.36	4.68	4.2	4.41	3.84
	95.62	74.84	76.94	78.64	73.18
→4)-α-L-Fuc-(2,3SO_3^-)-(1→3)- (**C**)	5.37	4.64	4.74	4.27	4.54
	99.5	73.27	74.79	80.22	68.8
→3)-α-L-Fuc-(2SO_3^-) (**B**)	5.5	4.53	4.06	4.09	4.22
	91.1	74.29	74.3	69.79	66.46

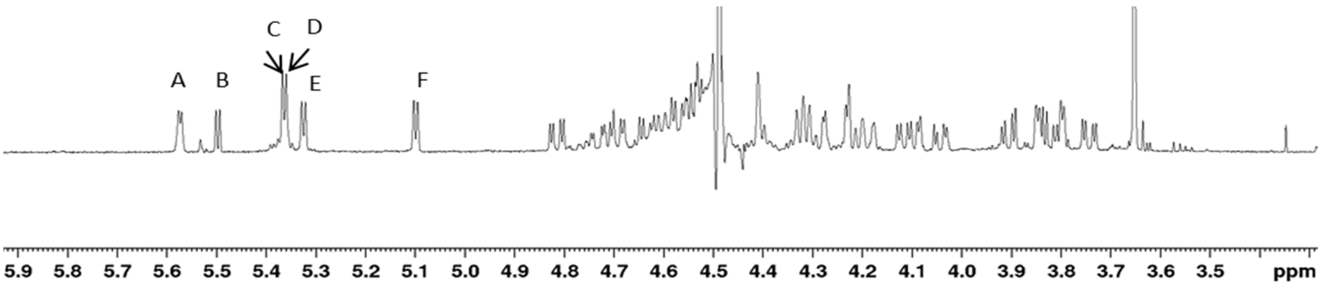

Figure S8. ^{1}H NMR spectra of the Oligo_fr1.

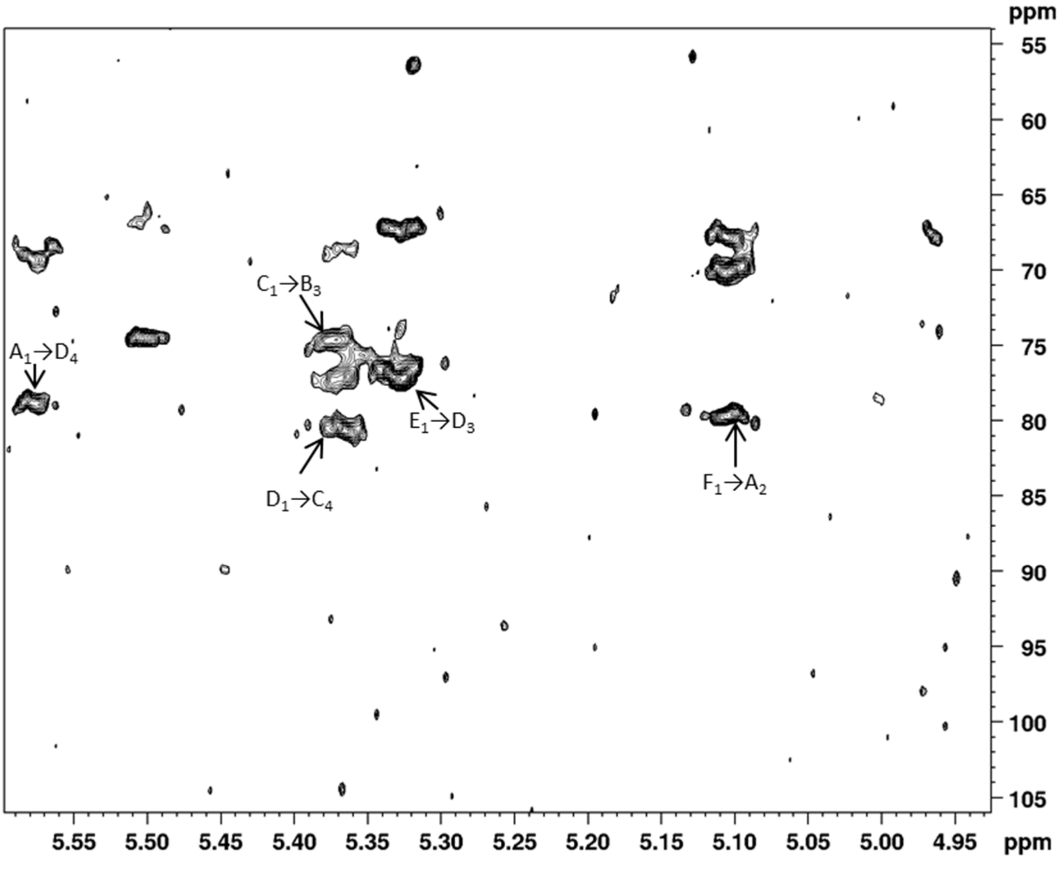

Figure S9. HMBC spectrum of the Oligo_fr1.

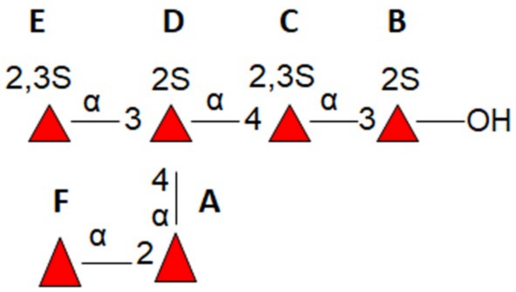

Figure S10. Structure of the Oligo_fr1.
